# Mussel‐Derived and Bioclickable Peptide Mimic for Enhanced Interfacial Osseointegration via Synergistic Immunomodulation and Vascularized Bone Regeneration

**DOI:** 10.1002/advs.202401833

**Published:** 2024-06-23

**Authors:** Wei Zhou, Yang Liu, Jiale Dong, Xianli Hu, Zheng Su, Xianzuo Zhang, Chen Zhu, Liming Xiong, Wei Huang, Jiaxiang Bai

**Affiliations:** ^1^ Department of Orthopaedics Union Hospital Tongji Medical College Huazhong University of Science and Technology Wuhan 430022 China; ^2^ Department of Orthopaedics The First Affiliated Hospital of USTC Division of Life Sciences and Medicine University of Science and Technology of China Hefei 230022 China

**Keywords:** bone regeneration, immunomodulatory, mussel adhesion, orthopedic implant, tissue adaptation

## Abstract

Inadequate osseointegration at the interface is a key factor in orthopedic implant failure. Mechanistically, traditional orthopedic implant interfaces fail to precisely match natural bone regeneration processes in vivo. In this study, a novel biomimetic coating on titanium substrates (DPA‐Co/GFO) through a mussel adhesion‐mediated ion coordination and molecular clicking strategy is engineered. In vivo and in vitro results confirm that the coating exhibits excellent biocompatibility and effectively promotes angiogenesis and osteogenesis. Crucially, the biomimetic coating targets the integrin α2β1 receptor to promote M2 macrophage polarization and achieves a synergistic effect between immunomodulation and vascularized bone regeneration, thereby maximizing osseointegration at the interface. Mechanical push‐out tests reveal that the pull‐out strength in the DPA‐Co/GFO group is markedly greater than that in the control group (79.04 ± 3.20 N vs 31.47 ± 1.87 N, *P* < 0.01) and even surpasses that in the sham group (79.04 ± 3.20 N vs 63.09 ± 8.52 N, *P* < 0.01). In summary, the novel biomimetic coating developed in this study precisely matches the natural process of bone regeneration in vivo, enhancing interface‐related osseointegration and showing considerable potential for clinical translation and applications.

## Graphical abstract

This study develops a novel biomimetic titanium coating 3,4‐dihydroxy‐L‐phenylalanine‐Co‐GFOGER/(DPA‐Co/GFO) that can target the integrin α2β1 receptor to promote M2 macrophage polarization, creating a favorable immune microenvironment conducive to osteogenesis and angiogenesis. This synergistic effect on immunomodulation and vascularized bone regeneration can precisely match natural bone regeneration in vivo, thereby facilitating high‐quality osseointegration at the bone–implant interface.

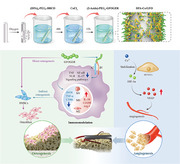

## 1 Introduction

The clinical application of orthopedic implants has achieved great success over the past several decades.^[^ ^1^ ^]^ However, traditional bone implants still have issues such as poor bioactivity and the inability to adequately adapt to complex physiological bone regeneration processes, resulting in insufficient integration of the bone‐to‐metal interface over the long term.^[^ ^2^, ^3^ ^]^ Moreover, as the global population ages, the increasing prevalence of conditions such as osteoporosis, diabetes, and chronic kidney disease further amplifies the challenges associated with orthopedic implant applications.^[^ ^4^, ^5^, ^6^ ^]^ An ideal bone implant can activate various cell factors in the body to modulate the regeneration and reconstruction of bone, thereby achieving stable binding to adjacent bone tissue.^[^ ^7^ ^]^ Since the interaction between the implanted material and the surrounding environment occurs over a minimal thickness at the surface, manipulating the properties of the surface interface can promptly and efficiently facilitate the functional compatibility of the material with adjacent tissues without compromising other characteristics of the substrate.^[^ ^8^ ^]^ Therefore, designing and constructing tissue‐tailored bone implant surface interfaces to precisely align with the particularities of the bone regeneration process has received considerable attention.

To date, the focus of research in this field has been on augmenting the osteogenic induction of stem cells by bone implant surfaces.^[^ ^9^ ^]^ However, despite promising results in vitro, some bone implant materials have been shown to be less effective in vivo.^[^ ^10^, ^11^ ^]^ The underlying reason for this difference is that bone repair mediated by foreign materials within the body involves a complex cascade of reactions involving multiple cell types and includes four interrelated and synergistic phases: hematoma formation/the inflammatory response, angiogenesis, new bone formation, and bone remodeling.^[^ ^12^ ^]^ During the early stages of healing, a hematoma rapidly develops on the surface of the implant, which activates the body's defense mechanisms, leading to the secretion of a broad array of mediators associated with inflammation by macrophages and various immune cells.^[^ ^13^ ^]^ During this period, macrophages are predominantly polarized toward the M1 phenotype, and their timely transition to the M2 phenotype is crucial for subsequent angiogenesis and osteogenesis mediated by endothelial cells (ECs) and bone marrow mesenchymal stem cells (BMSCs).^[^ ^14^ ^]^ Based on the above findings, an ideal bone implant material induces M2 polarization of macrophages postimmune activation, further stimulating vascularized bone regeneration and ultimately leading to the formation of a robust bond with peripheral bone tissue.

Hypoxia plays a critical role in in vivo angiogenesis by activating a series of angiogenic processes through HIF‐1α.^[^ ^15^, ^16^ ^]^ Cobalt, an essential trace element in the human body and a vital component of vitamin B12, has been proven to stabilize hypoxia‐inducible factor 1‐alpha (HIF‐1α), thereby inducing a cascade of angiogenic responses that efficiently promote local angiogenesis.^[^ ^17^ ^]^ Introducing cobalt ions to the modified titanium surface in this study is clearly a good choice. Recently, the construction of biomimetic extracellular matrix (ECM) components, particularly collagen, on implant surfaces has received extensive attention.^[^ ^18^, ^19^ ^]^ Gly‐Phe‐Hyp‐Gly‐Glu‐Arg (GFOGER), a triple‐helical short peptide from type I collagen, targets the integrin α2β1 receptor on cell membranes to enhance adhesion and differentiation.^[^ ^20^, ^21^ ^]^ Previous research has indicated that in addition to promoting cell adhesion, integrin α2β1 also contributes to immune regulation by promoting M2 polarization of macrophages.^[^ ^22^ ^]^ Therefore, developing targeted strategies for specific integrins, such as grafting GFOGER peptides onto the surfaces of implant materials to achieve direct regulation of macrophage differentiation, is an extremely attractive research objective in surface bioengineering. To date, a variety of physical and chemical surface modification methods, including layer‐by‐layer self‐assembly, acid etching, and ion doping, have been used to introduce diverse bioactive molecules to the surfaces of implants.^[^ ^23^, ^24^, ^25^ ^]^ However, current methods of physical or chemical modification still have numerous limitations, such as molecular leakage, overly rapid release, complicated procedures, and lack of long‐term activity.^[^ ^26^, ^27^, ^28^ ^]^ Therefore, there is growing interest in developing simple and effective surface treatment methods that can bind specific bioactive molecules and exhibit tissue adaptability.

Since its introduction by Lee in 2007, surface chemical modification via dopamine polymerization has been considered a pioneering approach, with subsequent biomimetic strategies inspired by marine mussel foot proteins (MFPs) gaining recognition as highly promising methods for surface modification.^[^ ^29^, ^30^ ^]^ These methods involve the use of the repetitive catechol‐containing amino acid 3,4‐dihydroxy‐L‐phenylalanine (DPA) to induce robust surface adhesion across a wide range of surfaces.^[^ ^31^ ^]^ In addition, the catechol group can be easily conjugated with biomolecules and spontaneously coordinated with diverse metal ions.^[^ ^32^, ^33^, ^34^ ^]^ The aforementioned benefits suggest that mussel‐inspired surface modification strategies have the potential to simultaneously modify bone implants with tissue‐adaptive inducer molecules and bioactive metal ions. In our previous work, we designed two mussel‐inspired biomimetic peptides, DOPA‐RGD and DOPA‐OGP, which enable simple modification of titanium substrates through catechol/TiO_2_ coordination, resulting in excellent osteoinductive and immunomodulatory activities.^[^ ^22^, ^35^ ^]^ Nonetheless, this strategy is suitable only for simple grafting due to uncontrolled molecular conjugation, and the random utilization of active functional groups such as ─NH_2_ and ─COOH during multiple modifications poses challenges for preserving molecular activity.^[^ ^36^ ^]^ Benefiting from the gentle and uncomplicated reaction settings, rapid reaction kinetics, selectivity, and biological compatibility, the emergence of click chemistry has ingeniously addressed this problem.^[^ ^37^, ^38^ ^]^ Based on these findings, we developed an improved biomimetic modification approach that integrates mussel‐inspired adhesion with bioorthogonal click reactions (azide–alkyne cycloaddition reactions). We propose that this advanced biomimetic modification strategy, which encompasses the bioorthogonal conjugation of active molecules and the coordination of ion loading, could offer a promising solution for multiple modifications.

**Figure 1:**
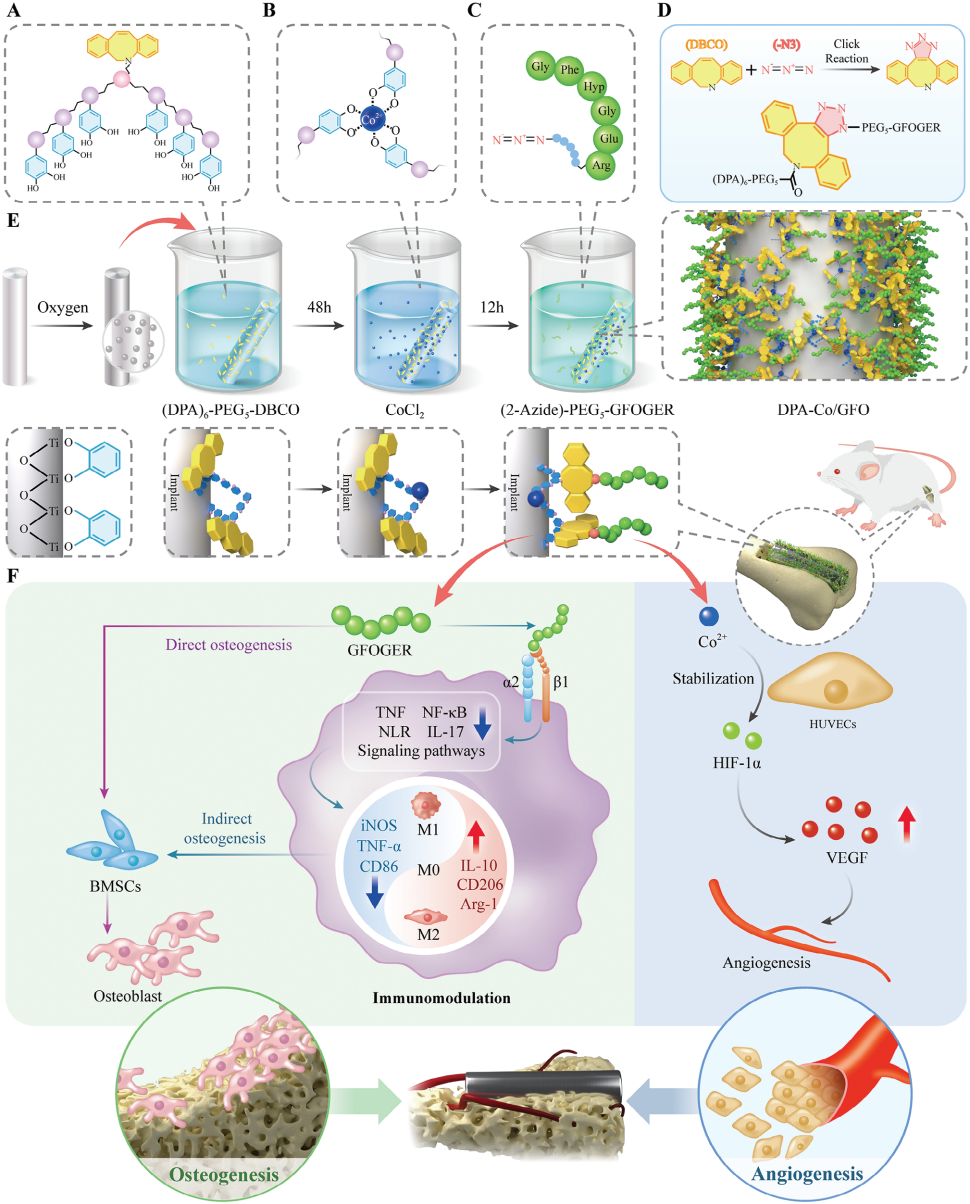
Schematic of the bone implant surface design tailored to the in vivo bone regeneration process. A) (DPA)_6_‐PEG_5_‐DBCO (Mussel derived peptide with a bioclickable DBCO group). B) Metal‐catechol coordination. C) 2‐Azido‐(PEG_5_)‐GFOGER (GFOGER peptide capped with an azide (─N_3_) group). D) Bioorthogonal click reactions (azide–alkyne cycloaddition reactions). E) Synthesis of a novel biomimetic titanium surface (DPA‐Co/GFO). F) Immunomodulatory synergy with vascularized bone regeneration.

In this work, a DPA‐containing peptide was synthesized by incorporating a bioclickable dibenzocyclooctyne (DBCO) group that was stably anchored to a titanium‐based surface via metal‒catechol coordination and subsequently incorporated an angiogenic cobalt ion (Co^2+^) to construct a vascularized regeneration platform. To regulate the directed differentiation of macrophages at the interface, we synthesized a GFOGER peptide capped with an azide group (─N_3_), conjugated it to the DBCO group via click chemistry, and ultimately created a novel biomimetic titanium surface (DPA‐Co/GFO) (**Figure** **1**A–F). After modification, we examined the regulatory effect of DPA‐Co/GFO on angiogenesis and osteogenesis in vitro and in vivo and further investigated the synergistic effects on immunomodulation and vascularized bone regeneration. In summary, our proposed surface modification strategy provides a simple and adaptable multifunctional modification method with tremendous potential for clinical applications. The novel biomimetic coating developed in this study is particularly suitable for clinical scenarios involving orthopedic or dental prostheses. This modified surface is able to harmonize the osteoimmune response and vascularized bone regeneration, thereby maximizing osteointegration at the interface.

## 2 Results and Discussion

### 2.1 Materials Synthesis and Surface Modification

**Figure 2:**
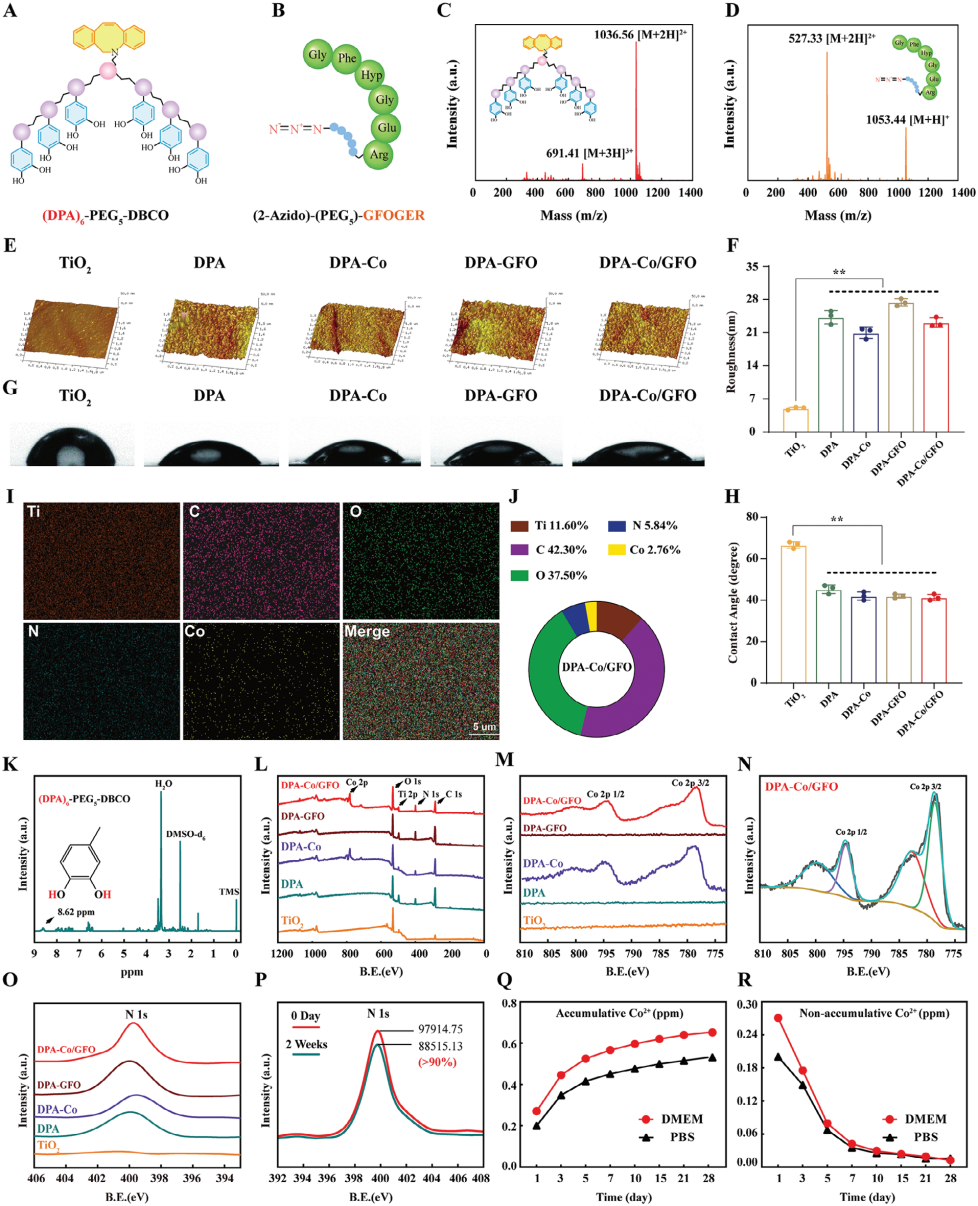
Material characterization of the different modified surfaces. A,B) Schematic representations of the (DPA)_6_‐PEG_5_‐DBCO and (2‐Azido)‐PEG_5_‐GFOGER structures. C,D) ESI‐MS spectra of (DPA)_6_‐PEG_5_‐DBCO and (2‐Azido)‐PEG_5_‐GFOGER. E,F) AFM images of different modified surfaces and quantification of surface roughness. G,H) Water contact angles on the different surfaces and the corresponding quantitative results. I,J) SEM‒EDS elemental mapping and quantitative analysis of the elemental composition of the surface modified with Co^2+^ and the GFOGER peptide (DPA‐Co/GFO). K) ^1^H NMR spectra of (DPA)_6_‐PEG_5_‐DBCO. L–O) XPS analysis of the different modified surfaces. P) Changes in the N 1 s signal within the XPS spectrum of the DPA‐Co/GFO surface following 2 weeks of incubation in DMEM. Q,R) Accumulative and nonaccumulative release curves of Co^2+^ from the DPA‐Co/GFO surface in PBS solution and DMEM. The data are presented as the mean ± standard deviation (SD) (*n* = 3 per group). Statistical analysis was performed by one‐way ANOVA, and *P < 0.05 and **P < 0.01 indicate statistical significance.

Solid‐phase peptide synthesis was used to synthesize mussel‐inspired peptides containing clickable DBCO groups following the methodology described in previous publications.^[^ ^35^, ^37^ ^]^ In this study, readily available Fmoc‐DPA (acetone)─OH was utilized to integrate DPA within the peptide chain. To guarantee a sufficient quantity of catechol structures for Co^2+^ coordination and DBCO structures for subsequent biomolecular click reactions following peptide attachment to titanium substrates, hexamer‐DPA structures and DBCO containing an extended polyethylene glycol (PEG) sequence were utilized in the synthesis of the peptide derived from mussels ((DPA)_6_‐PEG_5_‐DBCO) (**Figure** **2**A and Figure S1A, Supporting Information). Furthermore, the GFOGER peptide capped with an azide group (─N_3_) was synthesized (2‐Azido‐(PEG_5_)‐GFOGER) (Figure 2B and Figure S1B, Supporting Information). Indeed, the facile attachment of (2‐Azido)‐PEG_5_‐GFOGER to (DPA)_6_‐PEG_5_‐DBCO‐bound surfaces represents a versatile approach for altering surface characteristics. Our surface modification strategy is more economical and practical than the traditional physical approach of modifying the surface morphology of the coating.^[^ ^39^ ^]^ Moreover, our modification strategy is safer, more effective and more conducive to maintaining long‐term biological activity than conventional chemical treatments.^[^ ^40^ ^]^ Notably, multifunctional surface modification of implant coatings has become an important direction of development.^[^ ^41^ ^]^ In this study, utilizing a mussel adhesion‐mediated ionic combination and molecular click strategy provided a simpler and more general approach to multifunctional surface modification.

The peptides were subjected to purification using high‐performance liquid chromatography (HPLC), the results of which showed a purity exceeding 96% (Figure S2A,B, Supporting Information). The molecular structures of the molecules were further confirmed by electrospray ionization mass spectrometry (ESI‐MS). The monoisotopic masses [M + 2H]^2+^ of (DPA)_6_‐PEG_5_‐DBCO and (2‐Azido)‐PEG_5_‐GFOGER were established to be 1036.56 Da and 527.33 Da, respectively. The measured values aligned with the calculated molecular weights of 2070.13 Da and 1052.02 Da, as depicted in Figure 2C,D. First, titanium plates were coated with (DPA)_6_‐PEG_5_‐DBCO by immersion in a mussel‐derived peptide solution (0.01 mg mL^−1^), hereinafter referred to as DPA. Then, the surfaces coated with the mussel‐derived peptides were subsequently immersed in a CoCl_2_ solution (2 mg mL^−1^) to facilitate the coordination of catechol residues with Co^2+^, resulting in the formation of a Co^2+^‐loaded surface (referred to as DPA‐Co). Finally, (2‐Azido)‐PEG_5_‐GFOGER peptides were conjugated via bioorthogonal click chemistry and metal–catechol coordination to form complex surfaces with Co^2+^ and GFOGER peptides (referred to as DPA‐Co/GFO). In addition, a surface modified with GFOGER without Co^2+^ loading (referred to as DPA‐GFO) and an unmodified TiO_2_ surface (referred to as TiO_2_) were fabricated.

The roughness alterations of the modified surfaces were investigated through atomic force microscopy (AFM) (Figure 2E). The surface roughness increased substantially as a result of modifying (DPA)_6_‐PEG_5_‐DBCO and (2‐Azido)‐PEG_5_‐GFOGER, as evidenced by quantitative analysis (Figure 2F). Similarly, a significant increase in surface wettability was observed upon subsequent modification with Co^2+^ or GFOGER peptides, as depicted in Figure 2G,H. This improvement can be attributed to the hydrophilic nature of the Co^2⁺^ ions adhered to the surface, as well as to the specific amino acid sequence encoded by GFOGER. The chemical structure of the mussel‐inspired peptide was identified using ^1^H NMR, which revealed the presence of DPA units through a distinct peak at 8.62 ppm corresponding to catecholic hydrogens (Figure 2K). The efficacy of Co^2+^ modification was verified through the use of energy dispersive X‐ray spectrometry (EDS) and X‐ray photoelectron spectroscopy (XPS). EDS elemental mapping demonstrated the presence of cobalt distributed on the DPA‐Co/GFO surface (Figure 2I). Furthermore, quantitative analysis indicated that cobalt and nitrogen accounted for 2.76% and 5.84%, respectively, of the total atoms, thereby highlighting the effectiveness of Co^2+^ and GFOGER peptide co‐modification (Figure 2J). The surface elemental composition was further analyzed via XPS to verify the co‐modification of Co^2+^ and the GFOGER peptide. Signal peaks representing carbon, titanium, and oxygen were exclusively detected in the TiO_2_ group, whereas the surfaces of the DPA‐Co and DPA‐Co/GFO groups exhibited Co 2p 3/2 and Co 2p 1/2 signal peaks at 778.3 and 794.3 eV, respectively, as depicted in Figure 2L–N.

Additionally, a nitrogen 1 s signal (N 1 s) was detected in the DPA, DPA‐Co, DPA‐GFO, and DPA‐Co/GFO groups, with an increase in signal strength following further modification of the DPA surface with (2‐Azido)‐PEG_5_‐GFOGER (DPA‐GFO and DPA‐Co/GFO groups) (Figure 2O). The stability of the GFOGER‐modified surface was assessed by subjecting the DPA‐Co/GFO titanium plate to a 2 week incubation in DMEM at a controlled temperature of 37 °C. Based on the data presented in Figure 2P, the XPS analysis reveals a slight decrease of less than 10% in the strength of the N 1 s signal. These findings suggested that the immobilized GFOGER peptide is extremely stable, which can be attributed to the formation of covalent bonds through the use of click chemistry involving DBCO and azide groups. Furthermore, the quantification of Co^2+^ release from surfaces treated with both Co^2+^ and GFOGER in PBS and DMEM was performed using inductively coupled plasma‐atomic emission spectrometry (ICP‒AES; JY2000‐2, France). Co^2+^ demonstrated swift release within the initial five‐day period, subsequently accompanied by a notable decrease in the release rate and gradual attainment of stability, eventually resulting in an accumulated concentration of approximately 0.6 ppm after 4 weeks (Figure 2Q,R). This pattern is in alignment with previous literature,^[^ ^42^, ^43^ ^]^ in which the majority (approximately 80%) of Co^2+^ is released within the first 15 d, subsequently entering a stable state of extremely low concentration release after 30 d. According to previous reports, cobalt ions at concentrations less than 5 ppm did not induce toxic effects in either in vivo or in vitro studies, while cobalt ions at concentrations of approximately 1 ppm promoted optimal vascularized bone regeneration.^[^ ^44^ ^]^ Notably, the peak period of angiogenesis during bone regeneration occurs between 7 and 14 d postinjury,^[^ ^45^ ^]^ and the peak release of Co^2+^ from the modified coatings in this study coincides with this timeframe, approximately between days 7–10. The consistency of the time window may be crucial for modified surfaces to promote angiogenesis in vivo. In summary, the above results collectively demonstrate the successful co‐modification of TiO_2_ surfaces with Co^2+^ and GFOGER peptides, highlighting the potential of DPA‐Co/GFO to exhibit safe and sustained biological activity.

### 2.2 Biocompatibility Evaluation of the Modified Surfaces

**Figure 3:**
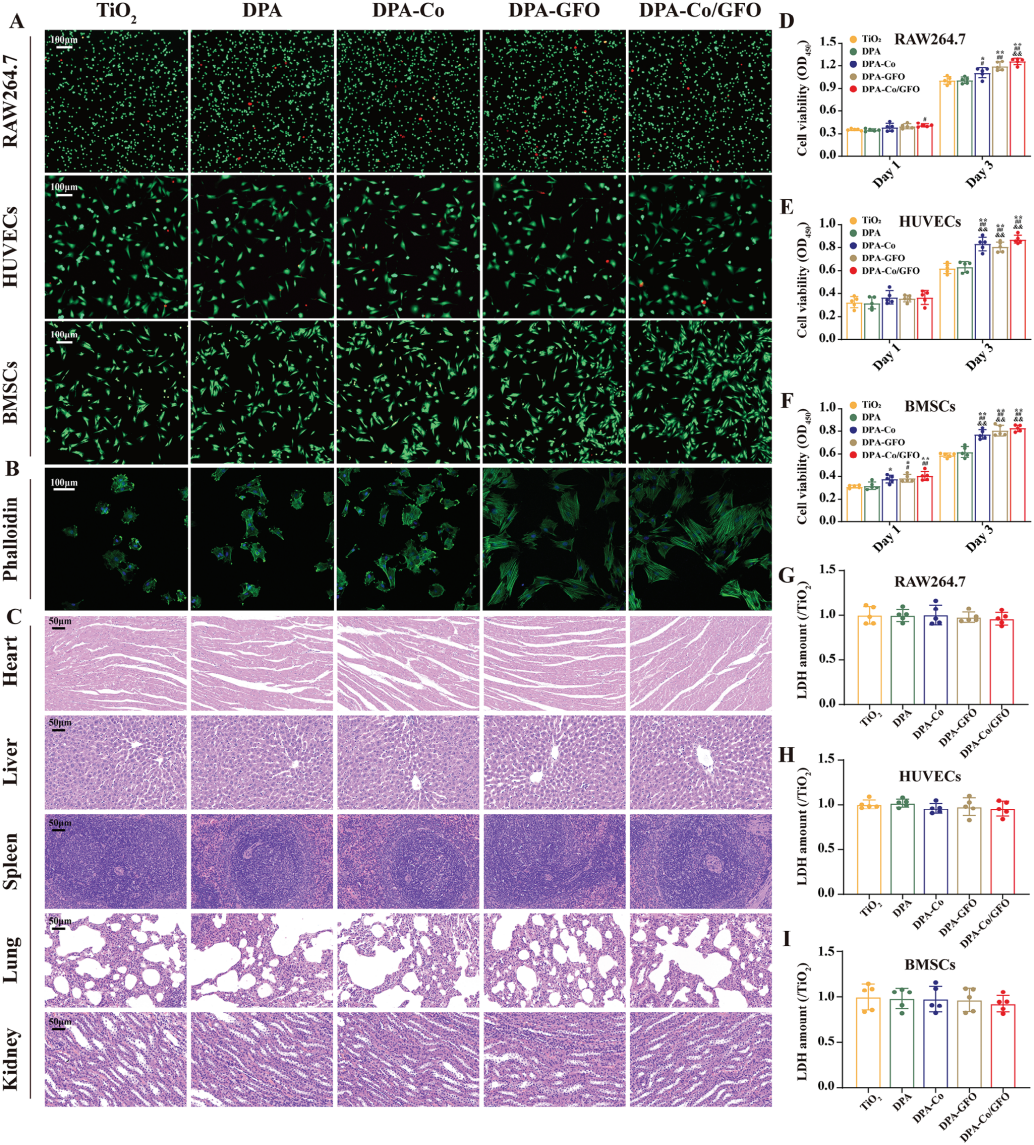
In vitro and in vivo biocompatibility of the different modified surfaces. A) Live/dead cell staining of RAW264.7 cells, HUVECs, and BMSCs on the different modified surfaces. B) Cytoskeletal staining (FITC‐phalloidin/DAPI) of BMSCs on the different modified surfaces. C) HE staining of rat viscera (heart, liver, spleen, lung, kidney) two months after the implantation of different modified titanium rods. D–F) CCK‐8 assays of RAW264.7 cells, HUVECs and BMSCs cultured on the different modified surfaces for 1 and 3 d. G–I) LDH cytotoxicity assays of RAW264.7 cells, HUVECs and BMSCs cultured on the different modified surfaces. The data are presented as the mean ± standard deviation (SD); *n* = 5 per group. Statistical analysis was performed by one‐way ANOVA (^∗^ *P* < 0.05 and ^∗∗^ *P* < 0.01 versus the TiO_2_ group; ^#^ *P* < 0.05 and ^##^ *P* < 0.01 versus the DPA group; ^&^ *P* < 0.05 and ^&&^ *P* < 0.01 versus the DPA‐Co group).

The primary prerequisite for all implant materials is biocompatibility.^[^ ^46^ ^]^ To evaluate the biocompatibility of the Co^2+^ or GFOGER‐modified surfaces, in vitro experiments were performed using RAW 264.7 cells, and live/dead cell staining assays were performed on HUVECs and BMSCs, revealing comparable numbers of dead cells across the DPA, DPA‐Co, DPA‐GFO, and DPA‐Co/GFO surfaces, with no significant differences from those on the unmodified TiO_2_ surface (**Figure** **3**A). The quantitative data confirmed these findings and demonstrated that the fraction of living cells on the different modified surfaces exceeded 90% (Figure S3A–C, Supporting Information). In addition, the morphological characteristics of adherent BMSCs on the different surfaces were examined through cytoskeleton staining (FITC‐phalloidin/DAPI) following a 24 h cultivation period. The results demonstrated that BMSCs exhibit a predominantly spherical morphology with minimal filopodia on TiO_2_ surfaces but exhibit polygonal shapes and robust expression of filamentous F‐actin on the DPA‐GFO and DPA‐Co/GFO surfaces (Figure 3B). These findings suggest that surfaces modified with GFOGER are more favorable for the adhesion and extension of BMSCs.

Furthermore, to evaluate the growth of RAW 264.7 cells, HUVECs, and BMSCs, the cell counting kit‐8 (CCK‐8) method was used. Compared with those of the other surfaces, the viability of the DPA‐Co, DPA‐GFO, and DPA‐Co/GFO surfaces increased across all three cell types (Figure 3D–F). Remarkably, the DPA‐Co/GFO surface demonstrated the highest level of cell proliferation, presumably attributable to the synergistic effect of Co^2+^ and GFOGER in enhancing cellular affinity. Moreover, the quantification of lactic dehydrogenase (LDH) released from RAW 264.7 cells, HUVECs, and BMSCs exposed to CO^2+−^ or GFOGER‐modified surfaces was conducted to evaluate cytotoxicity. After a 24 h culture period, the observed LDH levels in the aforementioned cell types were slightly lower than those in the bare TiO_2_ group, suggesting the absence of any cytotoxic effects (Figure 3G–I). Finally, different modified titanium rods were implanted into SD rats, and after two months, the major visceral organs of the rats were extracted for histological examination via hematoxylin and eosin (H&E) staining, revealing that the material also had no toxic effects on the animal organs (Figure 3C). Taken together, the results showed that surface modification with Co^2+^ and GFOGER peptides positively affects the growth of RAW 264.7 cells, HUVECs, and BMSCs, promoting cell proliferation and adhesion without significant cytotoxicity, suggesting the potential of DPA‐Co/GFO to establish an advantageous microenvironment conducive to bone regeneration.

### 2.3 Osteoimmunomodulation of the Modified Surfaces

**Figure 4:**
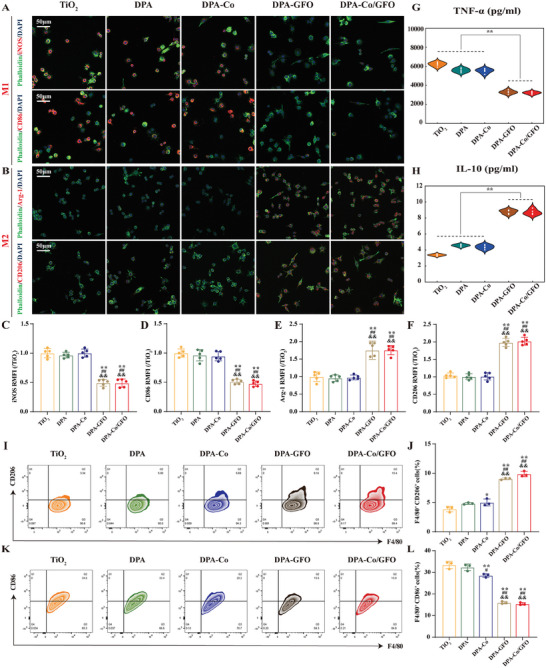
Regulation of macrophage polarization by the different modified surfaces in vitro. A,B) Immunofluorescence staining was used to evaluate macrophage polarization in RAW264.7 cells cultured on the different modified surfaces (green: phalloidin‐stained cytoskeleton; red: markers of M1 macrophages (CD86 and iNOS) and M2 macrophages (CD206 and Arg‐1); blue: nuclei). C–F) Quantitative results of immunofluorescence staining of the corresponding markers. G,H) ELISA was used to measure the secretion of the proinflammatory cytokine TNF‐α and the anti‐inflammatory cytokine IL‐10 by RAW264.7 cells cultured on the different modified surfaces. I–K) Flow cytometry was used to analyze the expression of CD86 (an M1 marker) and CD206 (an M2 marker) in RAW264.7 cells cultured on the different modified surfaces, and the results were quantified. The data are presented as the mean ± standard deviation (SD) (*n* = 3 or 5 per group). Statistical analysis was performed by one‐way ANOVA (^∗^ *P* < 0.05 and ^∗∗^ *P* < 0.01 vs the TiO_2_ group; ^#^ *P* < 0.05 and ^##^ *P* < 0.01 versus the DPA group; ^&^ *P* < 0.05 and ^&&^ *P* < 0.01 versus the DPA‐Co group).

Previous studies have shown that the presence of excess M1 macrophages following prosthetic implantation can lead to insufficient osteointegration at the interface, ultimately causing implantation failure.^[^ ^47^, ^48^ ^]^ Thus, the key to osteoimmunomodulation is the prompt conversion of inflammation‐inducing M1 macrophages to inflammation‐resolving M2 macrophages. The classically activated M1 phenotype stimulates inflammation through the production of TNF‐α, iNOS, and CD86, whereas the alternatively activated M2 phenotype, known for its healing properties, suppresses inflammation by secreting IL‐10, Arg‐1, and CD206.^[^ ^49^ ^]^ In this study, we initially performed immunofluorescence staining on RAW 264.7 cells to evaluate their polarization when cultured on various modified substrates. As illustrated in **Figure** **4**A–F, exposure to LPS resulted in an increase in M1 macrophages (marked by CD86^+^ and iNOS^+^; depicted in red) on the surfaces of the TiO_2_, DPA, and DPA‐Co groups. Conversely, on the surfaces of the DPA‐GFO and DPA‐Co/GFO groups, a greater proportion of M2 macrophages was observed (indicated by CD206^+^ and Arg‐1^+^, shown in red). Furthermore, cytokine secretion was assessed using an enzyme‐linked immunosorbent assay (ELISA). The results revealed that the levels of the proinflammatory cytokine TNF‐α were obviously greater in the TiO_2_, DPA, and DPA‐Co groups than in the DPA‐GFO and DPA‐Co/GFO groups (Figure 4G). In contrast, the DPA‐GFO and DPA‐Co/GFO groups exhibited significant increases in the production of the anti‐inflammatory cytokine IL‐10, as depicted in Figure 4H. These findings suggest that surface modification with the GFOGER peptide effectively stimulates macrophage activation toward the anti‐inflammatory M2 phenotype. Flow cytometry and Western blot analyses were also conducted. Notably, there was an increase in the ratio of F4/80^+^CD206^+^ cells (Figure 4I–J) as well as an increase in the expression of proteins associated with M2 phenotype polarization (CD206 and Arg‐1) (Figure S4A,D,E, Supporting Information) in the DPA‐GFO and DPA‐Co/GFO groups. Concurrently, a significant decrease in the ratio of F4/80^+^CD86^+^ cells (Figure 4K,L, Supporting Information) and M1 phenotype polarization‐related proteins (CD86 and iNOS) was noted (Figure S4A–C, Supporting Information). These results provide further support for the positive immunomodulatory effect of GFOGER‐modified surfaces in facilitating M2 phenotypic polarization.

**Figure 5:**
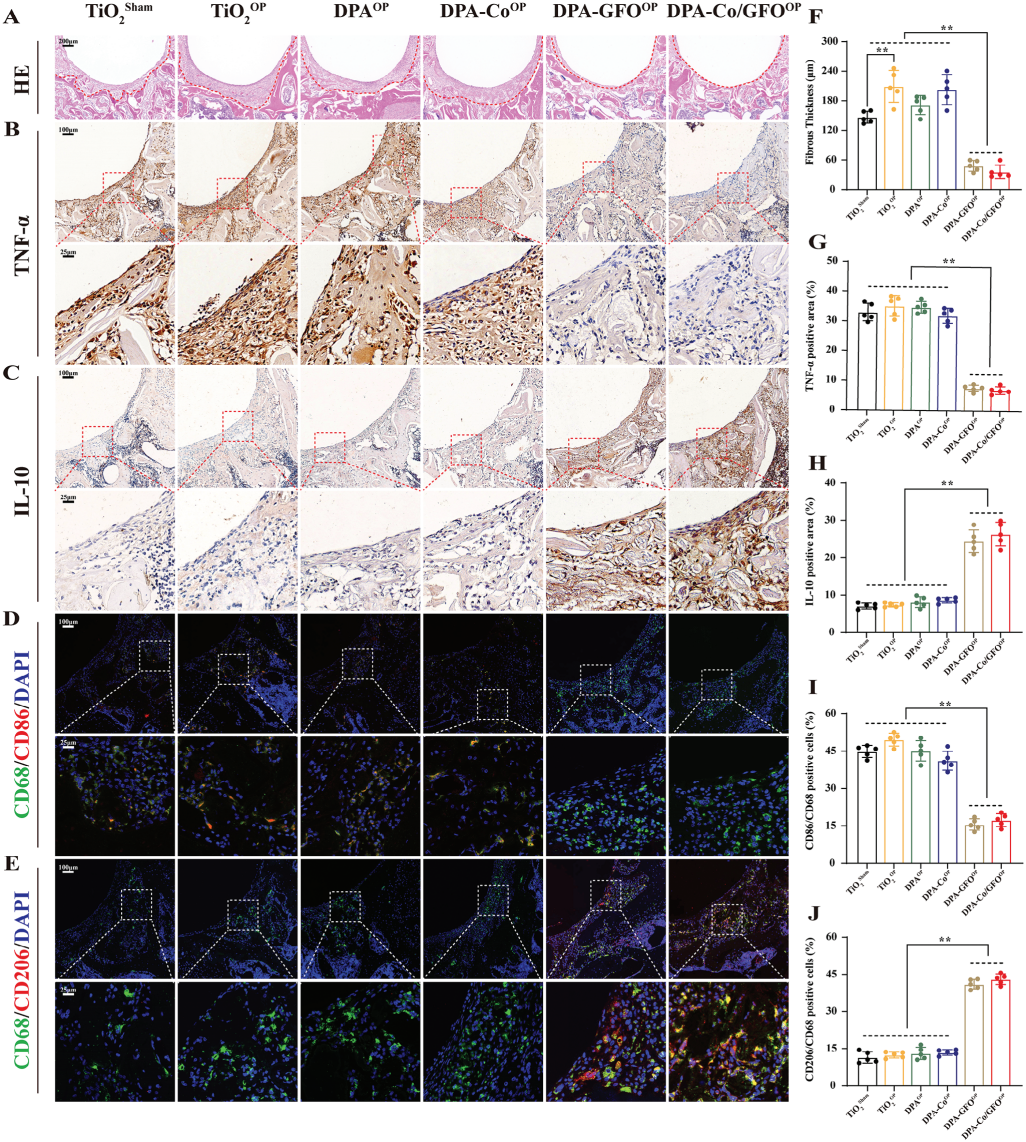
Regulation of macrophage polarization by the different modified surfaces in vivo. A) H&E staining of peri‐implant tissue in the femur 5 d after implantation and F) quantitative analysis of the thickness of the fibrous layer. B,C) Immunohistochemical staining of peri‐implant tissue to examine TNF‐α and IL‐10 expression, and G,H) the quantitative results of the corresponding positive areas are shown. D,E) Immunofluorescence staining of peri‐implant tissue to assess macrophage polarization status (green: macrophage‐specific marker (CD68); red: marker of M1 macrophages (CD86); and blue: nucleus of M2 macrophages (CD206)). I,J) The quantitative results are shown for positive cells. The data are presented as the mean ± standard deviation (SD) (*n* = 5 per group). Statistical analysis was performed by one‐way ANOVA, and *P < 0.05 and **P < 0.01 indicate statistical significance.

Previous in vitro experiments demonstrated the remarkable capacity of the DPA‐Co/GFO surface to guide the phenotypic conversion of macrophages from M1 to M2. To verify whether the same effect was observed in vivo, titanium rods with different surface modifications were implanted in the femurs of osteoporotic rats (from the TiO_2_^OP^, DPA^OP^, DPA‐Co^OP^, DPA‐GFO^OP^, and DPA‐Co‐GFO^OP^ groups), and bare titanium rods were implanted in nonosteoporotic (sham operation) rats as controls (from the TiO_2_^Sham^ group). Five days postimplantation, the rat femurs with the inserted titanium rods were dissected for histological evaluation via H&E staining. The increased thickness of the fibrous layer surrounding the prosthesis and the excessive penetration of inflammatory cells are commonly cited as prominent causes of implant failure.^[^ ^50^ ^]^ In this study, H&E staining revealed a reduced fibrous layer thickness and enhanced bone structure around the titanium rods in the rats in the DPA‐GFO^OP^ and DPA‐Co/GFO^OP^ groups compared to those in the other groups (**Figure** **5**A). Interestingly, compared with that in the TiO_2_^OP^ group, the fibrous layer in the TiO_2_^Sham^ group was significantly thinner, indicating that inflammatory dysregulation under osteoporotic conditions poses a greater challenge to the survival of the prosthesis than that under nonosteoporotic conditions. In addition, immunohistochemical staining further revealed a marked increase in the area positively stained for IL‐10 in the DPA‐GFO^OP^ and DPA‐Co/GFO^OP^ groups compared to that in the other groups (Figure 5C,H). In contrast, a significant decrease in the TNF‐α‐positive area was observed in the DPA‐GFO^OP^ and DPA‐Co/GFO^OP^ groups compared with the other groups (Figure 5B,G). Additionally, to further evaluate the phenotypic shift of macrophages surrounding the titanium rods, immunofluorescence staining was performed. Immunofluorescence imaging revealed a significantly greater number of cells expressing CD206, which is representative of M2 macrophages, in the DPA‐GFO^OP^ and DPA‐Co/GFO^OP^ groups than in the other groups (Figure 5E). The percentage of M2‐type macrophages in the DPA‐Co/GFO^OP^ group was approximately three times greater than that in the TiO_2_^OP^ group (Figure 5J). Conversely, the percentage of M1 macrophages that expressed CD86 in the TiO_2_^Sham^, TiO_2_^OP^, DPA^OP^ and DPA‐Co^OP^ groups was obviously greater than that in the DPA‐GFO^OP^ and DPA‐Co/GFO^OP^ groups (Figure 5D,I). In summary, both in vitro and in vivo findings indicate that surfaces modified with GFOGER may create a beneficial immune microenvironment, thereby promoting osteogenesis.

### 2.4 Immunomodulation‐Promoting Osteogenesis In Vitro

**Figure 6:**
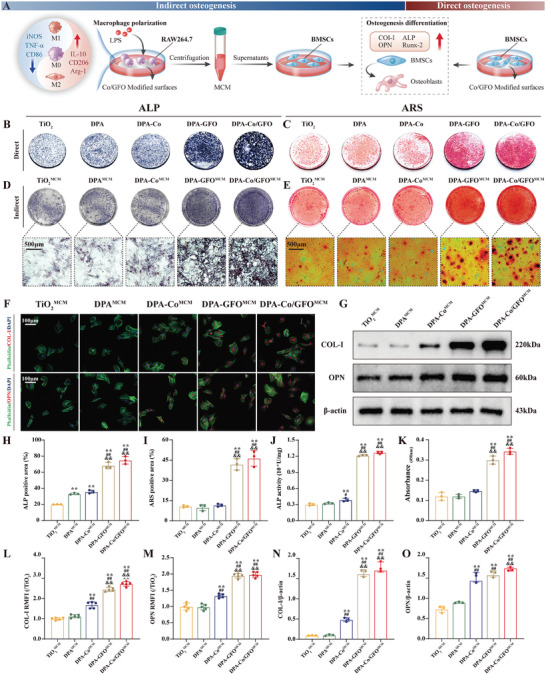
Direct and indirect osteogenic effects of GFOGER‐modified surfaces in vitro. A) Schematic diagram of the experimental design. B,C) BMSCs were cultured on different modified titanium surfaces, and ALP and ARS staining were used to assess the direct osteogenic effects. H,I) Quantitative analysis of the corresponding positive areas. D,E) BMSCs were cultured in macrophage‐conditioned medium (MCM) to investigate the indirect osteogenic effects (immunomodulation‐promoting osteogenesis) of the different modified surfaces, as shown by ALP and ARS staining, after which J,K) quantitative analysis was performed. F) Immunofluorescence staining was performed on BMSCs cultured in MCM, and the corresponding cells L,M) were subjected to quantitative analysis (green: phalloidin‐stained cytoskeleton; red: osteogenic markers (OPN and COL‐I); blue: nuclei). G) Western blot analysis of osteogenic markers and N,O) the quantitative results. The data are presented as the mean ± standard deviation (SD) (*n* = 3 or 5 per group). Statistical analysis was performed by one‐way ANOVA (^∗^ *P* < 0.05 and ^∗∗^ *P* < 0.01 vs the TiO_2_ group; ^#^ *P* < 0.05 and ^##^ *P* < 0.01 vs the DPA group; ^&^ *P* < 0.05 and ^&&^ *P* < 0.01 vs the DPA‐Co group).

The osteogenic potential, a critical factor in determining the degree of new bone formation on implant surfaces, is regulated not only by the physicochemical properties of the implant surface but also by the surrounding immune microenvironment.^[^ ^51^, ^52^ ^]^ Consequently, we further investigated whether surfaces modified with GFOGER could enhance osteogenic differentiation in vitro through an immunomodulatory effect using macrophage‐conditioned medium (MCM) (**Figure** **6**A). On day 7 of cell culture, alkaline phosphatase (ALP) staining revealed a notable increase in ALP activity in the DPA‐GFO^MCM^ and DPA‐Co/GFO^MCM^ groups compared to that in the TiO_2_^MCM^, DPA^MCM^, and DPA‐Co^MCM^ groups (Figure 6D). Quantitative analysis further substantiated the above results, showing that the ALP activity in the DPA‐GFO^MCM^ and DPA‐Co/GFO^MCM^ groups was approximately four times greater than that in the TiO_2_^MCM^ group (Figure 6J). Additionally, on day 21, Alizarin Red S (ARS) staining demonstrated that the DPA‐GFO^MCM^ and DPA‐Co/GFO^MCM^ groups exhibited mineral nodules that were approximately three times larger and more abundant than those of the TiO_2_^MCM^ group (Figure 6E,K). This observation suggested that calcium deposition is most efficient in MCMs derived from surfaces modified with GFOGER. Furthermore, the expression of osteogenic‐related proteins (COL‐I and OPN) was also determined through immunofluorescence staining and Western blot analysis. COL‐I and OPN expression in the DPA‐GFO^MCM^ and DPA‐Co/GFO^MCM^ groups was significantly greater than that in the TiO_2_^MCM^, DPA^MCM^, and DPA‐Co^MCM^ groups (Figure 6F,G,L–O). This further indicated that the MCMs derived from surfaces modified with GFOGER possess high potential for enhancing osteogenesis. In conclusion, the above findings suggest that surface modification with GFOGER enhances osteogenesis by facilitating the shift of macrophages from the M1 phenotype to the M2 phenotype, thereby creating a favorable immune microenvironment. Interestingly, surfaces modified with GFOGER also exhibited considerable direct osteogenic effects (Figure 6A). In this work, we cultured BMSCs directly on different modified surfaces and performed ALP and ARS staining on days 14 and 21, respectively. ALP‐ and ARS‐positive areas were significantly greater in the DPA‐GFO and DPA‐Co/GFO groups than in the other groups (Figure 6B,C,H,I). This result suggested that in addition to modulating the immune microenvironment to enhance osteogenesis, GFOGER‐modified surfaces may also directly activate osteogenesis‐related signaling pathways.

### 2.5 Release of Co^2+^ from Modified Surfaces Promotes Angiogenesis

**Figure 7:**
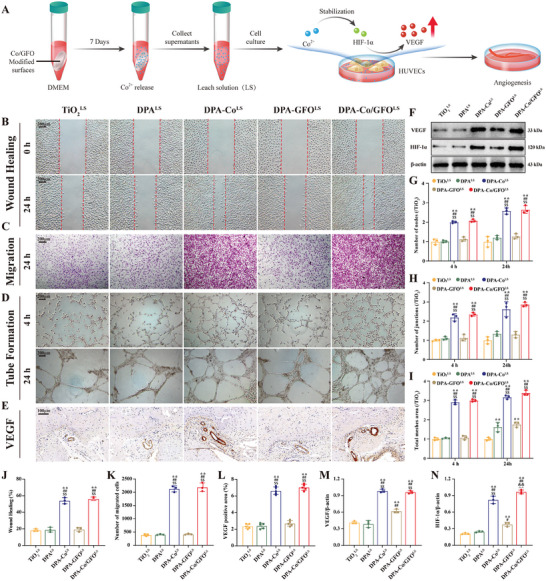
The release of Co^2+^ from modified surfaces (DPA‐Co and DPA‐Co/GFO) promotes angiogenesis. A) Schematic diagram of the experimental design. B) Wound healing assays were performed on HUVECs following 24 h of exposure to leach solutions from the different modified surfaces, and the corresponding G) quantitative data are shown. C) Transwell assays were performed on HUVECs after 24 h of incubation in leach solutions derived from different modified surfaces, and the corresponding K) quantitative data were collected. D) Tube formation assays were performed on HUVECs after 4 and 24 h of treatment with the leach solution, and the corresponding G–I) quantitative data were obtained. E) Immunohistochemical staining of the angiogenic marker VEGF in peri‐implant tissue and L) quantification of the corresponding positive area. Western blot analysis of osteogenic markers and N–O) the quantitative results. F) Western blot analysis of VEGF and HIF‐1α in the different groups and M,N) quantification results. The data are presented as the mean ± standard deviation (SD) (*n* = 3 or 5 per group). Statistical analysis was performed by one‐way ANOVA (^∗^ *P* < 0.05 and ^∗∗^ *P* < 0.01 versus the TiO_2_ group; ^#^ *P* < 0.05 and ^##^ *P* < 0.01 vs the DPA group; ^&^ *P* < 0.05 and ^&&^ *P* < 0.01 vs the DPA‐Co group; ^$^ *P* < 0.05 and ^$$^ *P* < 0.01 vs the DPA‐GFO group).

Angiogenesis has been acknowledged as a fundamental requirement for successful osseointegration at the bone‐implant interface.^[^ ^53^ ^]^ In this study, sterile titanium plates with various surface modifications were immersed in fresh high‐glucose DMEM for 7 d, after which the leached solution (LS) was collected for angiogenic activity analysis (**Figure** **7**A). First, the migratory ability of HUVECs was evaluated via a wound healing assay. After 24 hours, the rate of wound closure was markedly greater in both the DPA‐Co^LS^ and DPA‐Co/GFO^LS^ groups than in the other groups (Figure 7B). The quantitative analysis results confirmed that the wound healing rate in the DPA‐Co^LS^ and DPA‐Co/GFO^LS^ groups was approximately three times greater than that in the TiO_2_^LS^ group (Figure 7J). Moreover, a Transwell assay demonstrated that following a 24 h incubation period, there was a marked increase in cell migration in the DPA‐Co^LS^ and DPA‐Co/GFO^LS^ groups, while the number of migrated cells decreased in the other groups (Figure 7C,K). Subsequently, a tube formation assay was performed to observe vascularization. Similarly, after 4 and 24 h of incubation, the formation of nodes and junctions and the total mesh area increased significantly in the DPA‐Co^LS^ and DPA‐Co/GFO^LS^ groups, in sharp contrast to those in the TiO_2_^LS^, DPA^LS^, and DPA‐GFO^LS^ groups (Figure 7D,G–I).

Furthermore, we explored the angiogenesis‐related protein VEGF, which is a key factor governing neovascularization, to verify the outstanding angiogenic activity of the DPA‐Co^LS^ and DPA‐Co/GFO^LS^ groups. The results from both in vitro (WB) and in vivo (IHC) analyses demonstrated a noteworthy increase in VEGF expression in the cobalt‐modified group (DPA‐Co and DPA‐Co/GFO) compared to the other groups (Figure 7E,F,L,M). Overall, the above results suggest that the Co^2+^ released from the modified surfaces satisfactorily promotes angiogenesis. Studies have shown that cobalt ions can stabilize HIF‐1α, subsequently enhancing the production of VEGF and thereby stimulating angiogenesis.^[^ ^54^ ^]^ In this study, the protein levels of HIF‐1α were examined using Western blot analysis. Similarly, the results showed that the expression of HIF‐1α significantly increased in the cobalt‐modified group (DPA‐Co^LS^ and DPA‐Co/GFO^LS^) compared to that in the other groups (Figure 7F,N), which confirmed that the Co^2+^ released from the modified surfaces contributes to the promotion of angiogenesis.

### 2.6 Osteogenesis and Angiogenesis Synergistically Enhance Osseointegration In Vivo

**Figure 8:**
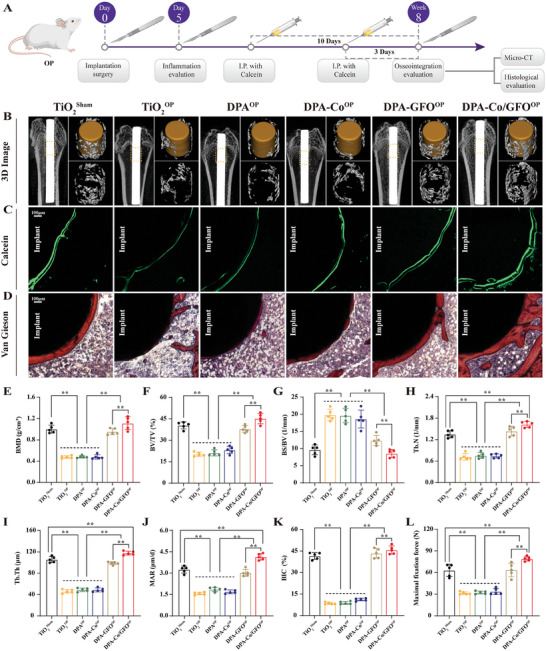
Evaluation of osseointegration around the different modified titanium rods. A) Animal experiment flowchart. B) Micro‐CT 3D reconstruction images and E–‐I) quantitative analysis of bone regeneration indices around the implant (BMD, BV/TV, BS/BV, Tb.​ N, and Tb.Th). C) Calcein fluorescence images of hard tissue sections and J) quantitative analysis of the mineral apposition rate (MAR). D) Van Gieson staining of hard tissue sections and K) quantitative analysis of the bone‐implant contact (BIC) are shown. L) Biomechanical pull‐out tests were performed to evaluate the maximum fixation force in the different groups. The data are presented as the mean ± standard deviation (SD) (*n* = 5 per group). Statistical analysis was performed by one‐way ANOVA, and *P < 0.05 and **P < 0.01 indicate statistical significance.

In vitro results demonstrated that the incorporation of GFOGER and cobalt surface modifications (DPA‐Co/GFO) can effectively enhance osteogenesis and angiogenesis. To further verify the synergistic effects of osteogenesis and angiogenesis in the DPA‐Co/GFO^OP^ group, which facilitated high‐quality osseointegration, related in vivo experiments were performed (**Figure** **8**A). The status of osseointegration at the interface between the titanium rod and bone was assessed 8 weeks after implantation. The 3D images reconstructed from micro‐CT scans revealed that, compared with those in the TiO_2_^Sham^, TiO_2_^OP^, DPA^OP^, DPA‐Co^OP^ and DPA‐GFO^OP^ groups, the DPA‐Co/GFO^OP^ group exhibited the greatest volume of fresh bone surrounding the titanium rod (Figure 8B). This result was further confirmed by quantitative analysis. The DPA‐Co/GFO^OP^ group demonstrated the highest bone mineral density (BMD) and bone volume fraction (BV/TV) and the lowest bone surface fraction (BS/BV) and exhibited the optimal characteristics of trabecular architecture (Tb. N and Tb. Th) (Figure 8E–I). Quantitative analysis revealed a 2.23‐fold increase in the BV/TV in the comodified DPA‐Co/GFO^OP^ group (45.18 ±3.58%) compared to that in the bare TiO_2_^OP^ group (20.25 ± 1.44%). Furthermore, the BV/TV of the comodified DPA‐Co/GFO^OP^ group was 1.95 and 1.20 times greater than that of the single DPA‐Co^OP^ (23.19 ± 2.51%) and DPA‐GFO^OP^ (37.97 ±2.41%) groups, respectively. Similarly, the trabecular number (Tb.N) and trabecular thickness (Tb.Th) in the DPA‐Co/GFO^OP^ group both improved by more than 120% compared to those in the DPA‐Co^OP^ group and by more than 13% compared to those in the DPA‐GFO^OP^ group. The DPA‐Co/GFO^OP^ group exhibited the most favorable integration of bone tissue with the implant, presumably as a result of the synergy between osteogenesis and angiogenesis. However, DPA‐Co^OP^ alone did not effectively promote osteogenesis, and DPA‐GFO^OP^ alone did not effectively stimulate neovascularization.

In addition, sequential fluorescence labeling conducted using calcein (green) to mark the newly formed bone revealed that the mineral apposition rate (MAR) of the DPA‐Co/GFOOP group was superior to that of the other groups (Figure 8C,J). Moreover, VG staining, Masson staining, and OPN immunohistochemistry were performed to further verify osseointegration at the interface. As expected, the DPA‐Co/GFO^OP^ group exhibited an optimal bone‐implant contact ratio (BIC), collagen volume fraction and OPN‐positive area (Figure 8D,K and Figure S5A–D, Supporting Information). Considering the direct correlation between the stability of the implant‐bone tissue interface and the success of clinical implantation procedures, we performed a biomechanical pull‐out test to evaluate the fixation strength of the titanium rod in the femur. As depicted in Figure 8L and Figure S6A–D (Supporting Information), the maximum pullout force of the DPA‐Co/GFO^OP^ group significantly increased compared to that of the other groups, suggesting superior mechanical stability. Specifically, the DPA‐Co/GFO^OP^ group exhibited the highest maximum pull‐out force (79.04 ± 3.20 N), which was 2.35 and 1.24 times greater than that of the DPA‐Co^OP^ (33.70 ± 4.26 N) and DPA‐GFO^OP^ (63.60 ± 9.49 N) groups, respectively.

The above results confirmed that the GFOGER and Co^2+^‐comodified surfaces promoted high‐quality osseointegration in vivo. In this work, we simultaneously incorporated the GFOGER peptide, known for its immunomodulatory and osteoinductive properties, and Co^2+^, recognized for its proangiogenic effects, into a modified surface. This multifunctional modification of the titanium surface is capable of synergistically promoting the osteoimmune response and the process of vascularized bone regeneration in vivo, thereby facilitating high‐quality osseointegration at the bone‐implant interface.

### 2.7 Comprehensive Analysis and Molecular Mechanism of Immunomodulation

**Figure 9:**
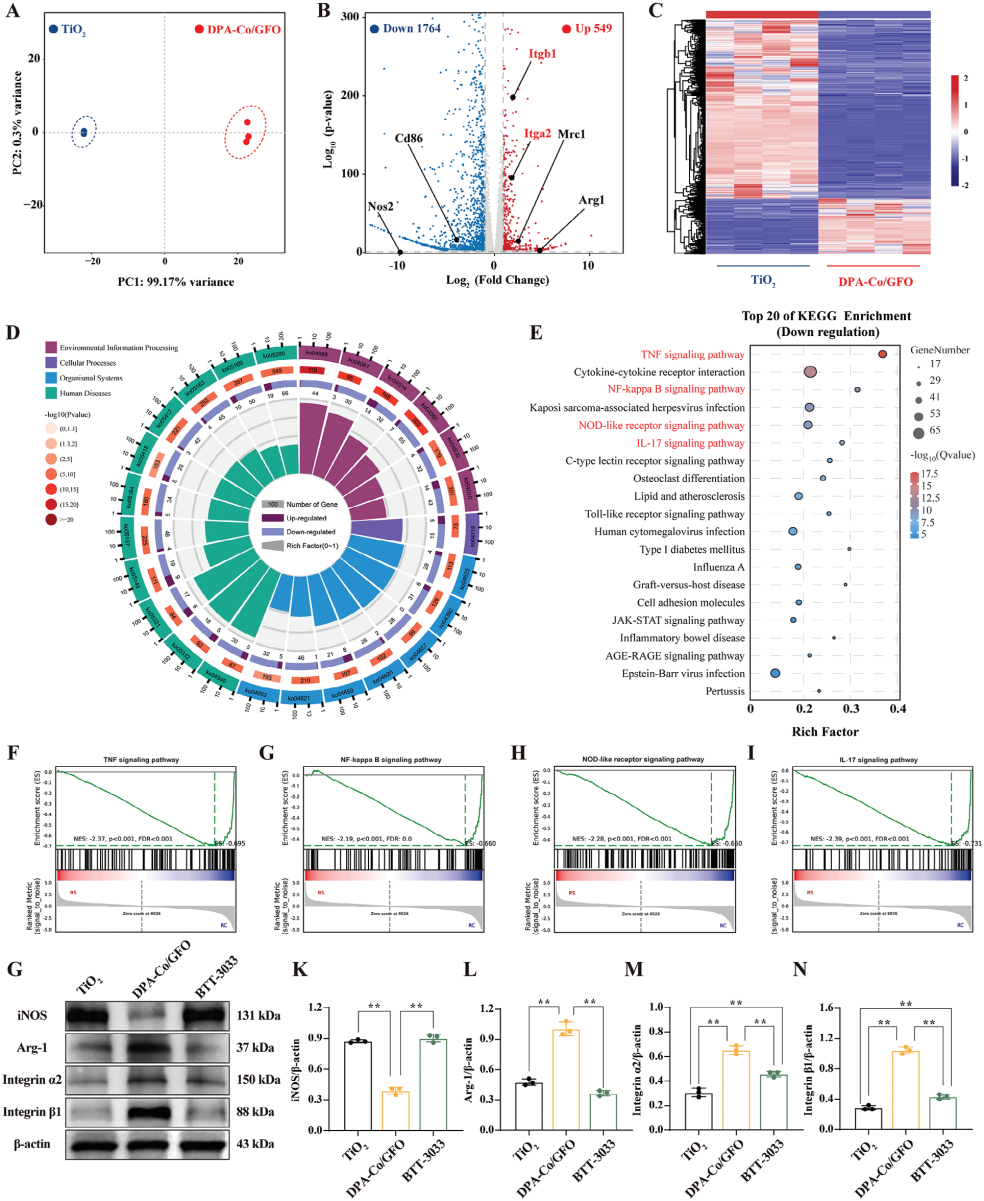
Transcriptome sequencing reveals the mechanisms by which DPA‐Co/GFO regulates M2 macrophage polarization. A) Principal component analysis (PCA) of DEGs in the TiO_2_ and DPA‐Co/GFO groups. B) Volcano plots of DEGs. C) Heatmap of DEGs. D) Gene Ontology (GO) enrichment analysis of DEGs in RAW264.7 cells cultured on DPA‐Co/GFO versus TiO_2_. E) The top 20 enriched pathways according to the Kyoto Encyclopedia of Genes and Genomes (KEGG) database (downregulation). F–I) Gene set enrichment analysis (GSEA) of the TNF, NF‐κB, NOD‐like, and IL‐17 signaling pathways. G) Western blot analysis to verify the role of DPA‐Co/GFO in targeting integrin receptor‐mediated regulation of M2 macrophage polarization (iNOS: M1 macrophage marker, Arg‐1: M2 macrophage marker, BTT‐3033: specific inhibitor of integrin α2β1). The data are presented as the mean ± standard deviation (SD) (*n* = 3 per group). Statistical analysis was performed by one‐way ANOVA, and *P < 0.05 and **P < 0.01 indicate statistical significance.

To achieve a deeper understanding of the immunoregulatory mechanism involved, RNA‐seq transcriptomic analysis was performed on RAW264.7 cells cultured on TiO_2_ and DPA‐Co/GFO surfaces. The results of principal component analysis (PCA) and component correlation analyses provided evidence that the samples satisfied the necessary criteria, thereby confirming the reliability of the RNA‐seq findings (**Figure** **9**A and Figure S7A–D, Supporting Information). A volcano plot of the DPA‐Co/GFO group relative to the TiO_2_ group revealed that 549 genes were upregulated and 1764 genes were downregulated (Figure 9B and Figure S8A, Supporting Information). The heatmap shows the differential expression of DEGs between the TiO_2_ and DPA‐Co/GFO groups, revealing that transcriptome reprogramming is significantly affected by DPA‐Co/GFO surface modification (Figure 9C). Gene ontology (GO) enrichment analysis of the DEGs indicated the significant involvement of the majority of these DEGs in modulating immune system responses (Figure 9D and Figure S8B, Supporting Information). Given the significant immunoregulatory differences highlighted by Gene Ontology (GO) analysis, we performed Kyoto Encyclopedia of Genes and Genomes (KEGG) analysis to identify the key immunoregulatory signaling pathways driving these functional changes. In the DPA‐Co/GFO group, the expression of TNF, NF‐κB (NF‐κB), NOD‐like receptor (NLR), and IL‐17 signaling pathway components was significantly downregulated (Figure 9E and Figure S8C, Supporting Information). Additionally, gene set enrichment analysis (GSEA) confirmed the significant downregulation of the expression of genes involved in these proinflammatory pathways in the DPA‐Co/GFO group (Figure 9F–I).

Upon mining the sequencing data, we discovered that, compared to those in the TiO_2_ group, genes associated with M1 polarization (NOS2 and Cd86) were downregulated, while genes related to M2 polarization (Mrc1 and Arg1) were upregulated in the DPA‐Co/GFO group, which aligns with the outcomes of our in vivo and in vitro experiments (Figure 9B). Notably, the upregulation of M2‐associated genes was accompanied by an increase in the expression of the integrin α2 (Itga2) and integrin β1 (Itgb1) genes, suggesting that the promotion of M2 polarization in the DPA‐Co/GFO group may be related to the activation of integrin α2 and integrin β1 receptors. To validate our hypothesis, we performed an immunofluorescence double‐staining experiment. The results demonstrated that in the DPA‐Co/GFO group, Arg‐1 expression (M2) was significantly upregulated concomitant with the upregulation of integrin α2 and integrin β1 expression (Figure S9, Supporting Information). Conversely, in the TiO_2_ group, a substantial upregulation of iNOS expression (M1) was observed, coupled with a considerable downregulation of integrin α2 and integrin β1 expression, aligning with our initial speculation. Furthermore, to show that the DPA‐Co/GFO‐modified surface facilitates macrophage M2 polarization through activation of the integrin α2β1 receptor, we used a specific inhibitor of integrin α2β1 (BTT‐3033) in a Western blotting experiment. Arg‐1 protein expression was significantly increased in the DPA‐Co/GFO group; however, this phenomenon was reversed after the addition of BTT‐3033 (Figure 9G–N), indicating that the DPA‐Co/GFO‐modified surface facilitates M2 macrophage polarization predominantly through the activation of the integrin α2β1 receptor.

In summary, the molecular mechanism underlying the immunomodulatory effect may be as follows: the GFOGER peptide within the DPA‐Co/GFO‐modified surface selectively targets and binds to the integrin α2β1 receptor on the macrophage membrane, thereby suppressing multiple proinflammatory signaling pathways (including the TNF, NF‐κB, NLR, and IL‐17 signaling pathways), which in turn promotes the shift in macrophages toward M2 polarization.

## 3 Conclusion

In summary, the biomimetic modification strategies presented in our study provide a straightforward and flexible approach for multifunctional surface modification. Through a mussel adhesion‐mediated ion coordination and molecular clicking strategy, we developed a novel biomimetic titanium surface (DPA‐Co/GFO) that regulated the directed differentiation of macrophages at the interface. This surface targeted the integrin α2β1 receptor, thereby inhibiting multiple downstream proinflammatory signaling pathways, promoting the shift in macrophages toward M2 polarization and creating a favorable immune microenvironment for subsequent osteogenesis and angiogenesis. The synergistic effect on immunomodulation and vascularized bone regeneration can be precisely matched with natural bone regeneration in vivo, thereby maximizing interface‐related osteointegration and holding great potential for further applications.

## 4 Experimental Section

### Coating Preparation

Two clickable peptide molecules were synthesized using the Fmoc solid‐phase synthesis method. Fmoc‐DPA (acetylonate)‐OH and Mal‐DBCO served as the raw materials for generating (DPA)_6_‐PEG_5_‐DBCO, while Fmoc‐NH‐PEG_5_‐CH_2_COOH and 2‐azidoacetic acid were used to synthesize (2‐Azido)‐PEG_5_‐GFOGER. Titanium plates (1 mm thick, 15 mm diameter; Tianjin Zhengtian Medical Devices Co., Ltd., China) and titanium rods (15 mm length, 1.5 mm diameter; Tianjin Zhengtian Medical Devices Co., Ltd., China) were meticulously cleaned by washing them four times with acetone, absolute alcohol, and ultrapure water. After drying, high‐temperature and high‐pressure sterilization were performed. To minimize catechol oxidation, the peptide solution was purged with nitrogen (N_2_) for 15 min before use. Then, the sterile titanium plates or rods were submerged in a phosphate‐buffered saline (PBS) solution containing 0.01 mg mL^−1^ PEG5‐(DPA)6‐DBCO for 48 h to achieve peptide coating. Next, the PEG_5_‐(DPA)_6_‐DBCO‐coated plates or rods were placed in a CoCl_2_ solution (2 mg mL^−1^) for 12 h. Finally, the Co^2+^‐loaded plates or rods were immersed in a PBS solution containing (2‐azido)‐(PEG_5_)‐GFOGER (1.0 × 10^−3^ m) for 12 h. The plates or rods comodified with Co^2+^ and GFOGER were meticulously washed with MiniQ water (18.2 MΩ cm) and subsequently dried using N_2_ in preparation for subsequent applications.

### Materials Characterization

The two synthetic peptides were purified using high‐performance liquid chromatography (HPLC), and subsequently, their molecular weights were determined through electrospray ionization mass spectrometry (ESI‐MS). With the aid of atomic force microscopy (AFM), the surface morphology of the titanium plates was evaluated for both modified and unmodified surfaces. Energy‐dispersive X‐ray spectroscopy (EDS) and X‐ray photoelectron spectroscopy (XPS) were used to determine the chemical makeup of the various specimens. The surface hydrophilicity of the various samples was evaluated using a Theta Lite contact angle measuring device (Biolin Scientific, Finland). The molecular structure was observed via nuclear magnetic resonance (NMR). Inductively coupled plasma‐atomic emission spectrometry (ICP‒AES, JY2000‐2, France) was used to analyze the release behavior of Co^2+^ from the DPA‐Co/GFO samples in PBS and DMEM.

### Cell Culture

RAW264.7 cells (ATCC, TCM13, Shanghai, China) were propagated in high‐glucose Dulbecco's modified Eagle's medium (DMEM, Gibco) supplemented with 10% fetal bovine serum (FBS) at a concentration of 100 units/mL penicillin/streptomycin. The cells were maintained in an incubator with a 5% CO_2_ atmosphere at 37 °C. The culture medium for the RAW264.7 cells was changed daily. Human umbilical vein endothelial cells (HUVECs) were obtained from the Cell Bank of the Shanghai Institute for Biological Sciences of the Chinese Academy of Sciences. These cells were cultured in high‐glucose DMEM supplemented with 10% FBS and 100 U/ml penicillin/streptomycin in a 5% CO_2_ environment at 37 °C. The culture medium used for HUVECs was refreshed every two days. In accordance with a previously established protocol,^[^ ^55^ ^]^ BMSCs (mesenchymal stem cells, harvested from bone marrow) were obtained from male Sprague Dawley (SD) rats aged 4 weeks from the Experimental Animal Center at Huazhong University of Science and Technology (HUST), located in Wuhan, China. In brief, the femur and tibia were meticulously dissected to detach them from the surrounding muscular and connective tissues. Low‐glucose DMEM supplemented with 10% fetal bovine serum (FBS) and 100 µ mL^−1^ penicillin/streptomycin was used to flush and suspend the bone marrow after both ends of the bone were removed. Subsequently, the cell suspension was strained through 70 µm mesh filters (Millipore, Ireland). Following filtration, the cells were cultured at 37 °C in an atmosphere containing 5% CO_2_, and the culture medium was replaced every two or 3 d. When the cells reached 80–90% confluence, they were detached from the culture dish by the application of 0.25% trypsin/EDTA solution.

### Biocompatibility

RAW264.7 cells, BMSCs, and HUVECs were separately cultured on samples that had undergone various surface treatments for a period of 24 h. Cell viability was evaluated using a kit for live/dead cell staining obtained from Yeasen, China. A Zeiss fluorescence microscope (Germany) was used to capture fluorescence images. The cytotoxicity of various modified surfaces and their effects on cell proliferation were evaluated through CCK‐8 (Yeasen, China) and LDH (Beyotime, China) assays. The attachment and structural characteristics of BMSCs on different surfaces were analyzed by chemoskeleton staining with phalloidin (Yeasen, China). The fluorescence images of the cells were observed using laser confocal microscopy (Zeiss, LSM800, Germany).

### In Vitro Macrophage Polarization

To assess the influence of surfaces modified with GFOGER on macrophage polarization, RAW264.7 cells were seeded at a density of 2 × 10^4^ cells per well across different surfaces in a 24‐well culture plate. Subsequently, the cells were stimulated with 100 ng mL^−1^ lipopolysaccharide (Sigma‒Aldrich) for 8 h, which facilitated the induction of the M1 phenotype. After three washes with PBS, the cells were incubated in fresh high‐glucose DMEM supplemented with 10% FBS. Following a culture period of 48 h, supernatants from the medium were collected to assess the secretion of TNF‐α and IL‐10 utilizing commercially available ELISA kits (ELK Biotechnology, ELK1387 and ELK1143). Additionally, immunofluorescence staining was used to observe distinct macrophage surface markers across different samples, with the aim of investigating macrophage polarization. The specimens were washed three times and then fixed with 4% paraformaldehyde (PFA; Sangon Biotech) for 20 min. Subsequently, permeabilization was performed using 0.1% (v/v) Triton X‐100 (Sigma) for a period of 10 min. To prevent nonspecific binding, the specimens were blocked with 2% bovine serum albumin (BSA; Sigma) for 1 h. Subsequently, the samples were incubated with primary antibodies at 4 °C overnight. In this research, the primary antibodies used were against CD86 (Immunoway, catalog YT7823), iNOS (Abcam, catalog ab178945), CD206 (Abcam, catalog ab64693), and Arg‐1 (Abcam, catalog ab91279). The specimens were subsequently incubated with Phalloidin‐iFluor 488 (green; Abcam, ab176753) and goat anti‐rabbit IgG H&L (Alexa Fluor 647, red; Abcam, ab150079) at 37 °C for 1 h. DAPI was used for nuclear staining, after which the sample was imaged with a Zeiss LSM800 laser confocal microscope. For semiquantitative analysis, three coverslips per group were used, and within each group, three separate subregions were selected at random. The positively stained cells and images were analyzed using ImageJ software (version 1.54d). Macrophage polarization was assessed using flow cytometry. Briefly, cells from different groups were harvested and subjected to staining with F4/80 (Thermo, 11‐4801‐82), CD206 (Thermo, 17‐2061‐80), and CD86 (Thermo, 12‐0862‐81) antibodies for 30 min. After two subsequent washes, the M1 (F4/80+/CD86+) and M2 (F4/80+/CD206+) macrophage subsets were identified utilizing flow cytometry (Thermo Scientific, USA), and subsequent data analysis was subsequently conducted using FlowJo V10 software.

### Osteogenic Differentiation In Vitro

To investigate whether GFOGER‐loaded substrates affect BMSC differentiation by modulating macrophage polarization, we collected supernatants from RAW264.7 cells cultured on various surfaces. Subsequently, these supernatants were mixed in equal ratios with fresh low‐glucose DMEM to generate macrophage‐conditioned medium (MCM). BMSCs were seeded at a density of 2 × 10^4^ cells per well and incubated in low‐glucose DMEM for 12 h. Subsequently, the initial medium was exchanged with macrophage‐conditioned medium (MCM) supplemented with osteogenic factors, including 10 × 10^−3^ m β‐glycerophosphate, 0.1 × 10^−6^ m dexamethasone, and 0.25 × 10^−3^ ascorbate, to continue the culture process. On day 7, ALP staining and activity were measured, while on day 21, ARS staining and quantitation were performed using osteogenic staining and quantification methodologies identical to those previously described. The expression levels of two osteogenesis‐related proteins, OPN (Immunoway, YT3467) and COL‐I (Abcam, ab270993), were evaluated through the application of immunofluorescence staining. Simultaneously, to confirm the direct osteogenic influence of the GFOGER‐loaded substrate, BMSCs were cultured directly on various surfaces in low‐glucose DMEM, and ALP and ARS staining were performed at the indicated time points.

### Angiogenesis In Vitro

To investigate whether Co^2⁺^‐loaded substrates promote angiogenesis in HUVECs, sterile titanium plates with various surface modifications were immersed in fresh high‐glucose DMEM for 7 d, after which the supernatants were collected as the leached solution (LS). HUVECs were seeded at a density of 1 × 10^6^ cells per well and incubated for 24 h in high‐glucose DMEM within a six‐well plate. A uniform scratch was then made across the well diameter in each well, and the medium was replaced with LS. The scratch ability was assessed at 0 and 24 h using a microscope (Zeiss, Germany). A cell migration assay was conducted using a Transwell system (Corning, USA). HUVECs were seeded in the upper chamber at a density of 2 × 10^3^ cells/200 µL, while LS was placed in the bottom chamber. After incubating for 24 h, the upper compartments were washed and stained with a 0.1% crystal violet solution (Solarbio, China). Images were captured using a microscope (Zeiss, Germany). Finally, a tube formation assay was performed. A combination of ABW Matrigel matrix and serum‐free DMEM in a 2:1 ratio was prepared, and 50 µL of this mixture was added to each individual well of a 96‐well plate. Subsequently, the mixture was incubated at 37 °C for 40 min. HUVECs were cultured at a density of 4 × 10^5^ cells per well and cultured with LS. The cells were cultured at 37 °C with a normal oxygen supply, and the tube formation results for HUVECs at 4 and 24 h were visualized using a microscope (Leica, Germany) and analyzed with ImageJ (version 1.54d).

### Western Blot Analysis

Total proteins were isolated from RAW264.7 cells, BMSCs and HUVECs following various treatments and quantified. The harvested proteins were electrophoresed via SDS‒PAGE for 90 min at 110 V, followed by transfer to PVDF membranes at 350 mA for 30 min. After blocking, primary antibodies against CD86 (Immunoway, YT7823), iNOS (Abcam, ab178945), CD206 (Abcam, ab64693), Arg‐1 (Abcam, ab91279), COL‐I (Abcam, ab270993), OPN (Immunoway, YT3467), VEGF (Immunoway, YN5444), HIF‐1α (Proteintech, 66730‐1‐lg), integrin α2 (HUABIO, ET1611‐57), integrin β1 (Affinity Biosciences, AF5379) and β‐actin (Immunoway, YM3028) were incubated with the sections at 4 °C overnight. After being rinsed extensively with TBST solution, the PVDF membrane was incubated for 1 h with the appropriate secondary antibody. Quantification was performed using ImageJ software (version 1.54d).

### Animal Models

All the animals in this study were obtained from the Experimental Animal Center of Tongji Medical College. The animal experiments were conducted in strict compliance with the ethical standards and protocols established by the Animal Care and Use Committee of Tongji Medical College (ethics approval number: [2022] IACUC Number: 3625). Ninety Sprague–Dawley rats (SD, female, 6–8 weeks old) were anesthetized by intraperitoneal injection of pentobarbital (nembutal, 3.5 mg/100 g), and bilateral ovariectomy or a sham operation was performed. Two months later, the bone mineral density (BMD) of each rat was ascertained through dual‐energy X‐ray absorptiometry (DEXA; Lunar Corporation, Madison, WI, USA), with the aim of evaluating the successful establishment of the osteoporosis model. Following the successful establishment of the osteoporosis model, 75 osteoporotic (OP) rats and 15 sham‐operated rats were divided into six groups (TiO_2_^Sham^, TiO_2_^OP^, DPA^OP^, DPA‐Co^OP^, DPA‐GFO^OP^, and DPA‐Co/GFO^OP^). As previously described,^[^ ^56^ ^]^ 15 rats per group underwent bilateral intramedullary titanium rod implantation in the distal femur (Figure S10A–E, Supporting Information). Five days after the implantation procedure, the experimental rats (five in each group) were euthanized, and the bilateral femurs containing the titanium rods were harvested for assessment of inflammation. The remaining rats (*n* = 10 per group) were injected intraperitoneally (i.p.) with 10 mg kg^−1^ calcein (Sigma) 10 and 3 d before euthanasia. Two months after the implantation surgery, the rats were euthanized, and their bilateral femurs, as well as their heart, liver, spleen, lung, and kidney, were collected for subsequent studies.

### Micro‐CT Analysis and Biomechanical Push‐Out Experiment

All femurs obtained two months after surgery were subjected to micro‐CT (Skyscan 1176, Belgium) scans. Each femur was scanned at a resolution of 18 µm per layer, with X‐ray parameters set at 50 kV and 500 µA, alongside a 0.7° rotational step. The bone mineral density (BMD, g cm^−3^), bone volume per tissue volume (BV/TV, %), bone surface/bone volume (BS/BV, 1 mm^−1^), trabecular number (Tb.N, 1 mm^−1^), and trabecular thickness (Tb.​Th, µm) were analyzed with CTAn software (Bruker, Belgium). Analysis of 3D models was conducted using Mimics Medical 21.0 software. Subsequently, half the left femurs (*n* = 5 per group) were subjected to biomechanical push‐out experiments to determine the peak load‐bearing capacity of the samples (Figure S6A–D, Supporting Information). The implant's maximum push‐out force was examined using a material testing system (HY1080, China). Prior to the biomechanical push‐out test, dental cement was used to anchor the femur containing the implant. The sample was fixed perpendicular to the bottom plane to ensure that the pushing force was parallel to the long axis of the implant. Afterward, the implant was continuously pushed along the loading direction at a velocity of 1 mm per minute. During the push‐out test, the force load is recorded to identify the maximum fixation strength.

### Histological Analysis

The left femurs (five from each group) were selected and dehydrated in 70% ethanol. These femurs were then utilized for undecalcified bone slicing. All fluorescence‐labeled bone sections were visualized using a fluorescence microscope (Zeiss, Germany). In addition, the slices were stained with van Gieson's stain and examined using an optical microscope (manufactured by Nikon, Japan) to assess the bone‐implant contact (BIC). Ten specimens from the right femoral bones of each group were collected and decalcified in 10% ethylenediaminetetraacetic acid at 37 °C for 5 weeks. Afterward, the titanium rods were carefully removed, and the femur was dehydrated and embedded in paraffin wax. Histological sections were prepared using a Leica 2135 microtome (Leica, Germany). Immunohistochemical analysis using OPN (Immunoway, YT3467) and Masson staining were performed to assess osteointegration. The femurs (*n* = 10 per group) collected five days after surgery were also sectioned into paraffin sections following the above method. H&E staining was used to assess inflammation around the implants. To assess macrophage polarization around the implants, immunofluorescence staining for CD68 (a macrophage marker; Abcam, ab201340), CD86 (an M1 marker; Immunoway, YT7823), and CD206 (an M2 marker; Abcam, ab64693) was performed. Additionally, immunohistochemical staining was conducted for TNF‐α (Servicebio, GB11188) and IL‐10 (Servicebio, GB11534). Semiquantitative analysis was performed using ImageJ software (version 1.54d).

### RNA‐seq and Data Analysis

RAW264.7 cells were cultured on TiO_2_ and DPA‐Co/GFO substrates. After 2 d of cultivation, total RNA was extracted, and its purity was assessed. RNA integrity was evaluated using an Agilent 2100 Bioanalyzer (Agilent Technologies, USA). Subsequently, a VAHTS Universal V6 RNA‐seq Library Prep Kit was used to construct the libraries following the manufacturer's guidelines. Transcriptome sequencing and analysis were performed by OE Biotech Co., Ltd. (China).

### Statistical Analysis

Outliers were identified and excluded based on Z scores, with a threshold set at ±3 standard deviations. The data are presented as the means ± standard deviations of at least three replicates for each experimental sample. Student's t test was used to verify the significance of differences between two groups, and one‐way ANOVA was performed for multiple comparisons. The results were considered statistically significant (*) when p<0.05 and highly significant (**) when *p* < 0.01. GraphPad Prism version 8.0 was used for all the statistical analyses.

## Conflict of Interest

The authors declare no conflict of interest.

## Supporting information

Supporting Information

## Data Availability

Research data are not shared.

## References

[1] R. J. Ferguson , A. J. Palmer , A. Taylor , M. L. Porter , H. Malchau , S. Glyn‐Jones , Lancet 2018, 392, 1662. 30496081 10.1016/S0140-6736(18)31777-X

[2] L. Qiu , Z. Zhu , F. Peng , C. Zhang , J. Xie , R. Zhou , Y. Zhang , M. Li , ACS Omega 2022, 7, 12030. 35449902 10.1021/acsomega.2c00229 PMC9016885

[3] F. A. D. Monte , K. R. Awad , N. Ahuja , H. K. W. Kim , P. Aswath , M. Brotto , V. G. Varanasi , Tissue Eng., Part A 2020, 26, 15. 31044666 10.1089/ten.tea.2019.0051 PMC6983748

[4] J. R. Corcuera‐Flores , A. M. Alonso‐Domínguez , M. Serrera‐Figallo , D. Torres‐Lagares , L. Castellanos‐Cosano , G. Machuca‐Portillo , J. Periodontol. 2016, 87, 14. 26334497 10.1902/jop.2015.150229

[5] F. Takeshita , K. Murai , S. Iyama , Y. Ayukawa , T. Suetsugu , J. Periodontol. 1998, 69, 314. 9579617 10.1902/jop.1998.69.3.314

[6] W. Liu , N. Kang , D. Seriwatanachai , Y. Dong , L. Zhou , Y. Lin , L. Ye , X. Liang , Q. Yuan , Sci. Rep. 2016, 6, 23041. 26955758 10.1038/srep23041 PMC4783709

[7] J. Sun , Y. Huang , H. Zhao , J. Niu , X. Ling , C. Zhu , L. Wang , H. Yang , Z. Yang , G. Pan , Q. Shi , Bioact. Mater. 2022, 9, 1. 34820551 10.1016/j.bioactmat.2021.10.003 PMC8586442

[8] W. Yu , H. Zhang , A. Lan , S. Yang , J. Zhang , H. Wang , Z. Zhou , Y. Zhou , J. Zhao , Z. Jiang , Colloids Surf., B 2020, 193, 111098. 10.1016/j.colsurfb.2020.111098 32498001

[9] J. Bai , G. Ge , Q. Wang , W. Li , K. Zheng , Y. Xu , H. Yang , G. Pan , D. Geng , Research 2022, 2022, 9823784. 36157511 10.34133/2022/9823784 PMC9484833

[10] Z. Chen , J. Yuen , R. Crawford , J. Chang , C. Wu , Y. Xiao , Biomaterials 2015, 61, 126. 26001077 10.1016/j.biomaterials.2015.04.044

[11] Y. Liu , L. Wang , T. Kikuiri , K. Akiyama , C. Chen , X. Xu , R. Yang , W. Chen , S. Wang , S. Shi , Nat. Med. 2011, 17, 1594. 22101767 10.1038/nm.2542 PMC3233650

[12] Y. Wang , Y. Zhang , R. J. Miron , Clin. Implant Dent. Relat. Res. 2016, 18, 618. 25873299 10.1111/cid.12343

[13] Q. Gu , H. Yang , Q. Shi , J. Orthop. Transl. 2017, 10, 86. 10.1016/j.jot.2017.05.002 PMC5822954 29662760

[14] B. N. Brown , S. F. Badylak , Acta Biomater. 2013, 9, 4948. 23099303 10.1016/j.actbio.2012.10.025

[15] O. Y. Fu , M. F. Hou , S. F. Yang , S. C. Huang , W. Y. Lee , Anticancer Res. 2009, 29, 3131. 19661326

[16] G. Basini , F. Grasselli , S. Bussolati , L. Baioni , F. Bianchi , M. Musci , M. Careri , A. Mangia , Steroids 2011, 76, 1433. 21827779 10.1016/j.steroids.2011.07.012

[17] C. Wu , Y. Zhou , W. Fan , P. Han , J. Chang , J. Yuen , M. Zhang , Y. Xiao , Biomaterials 2012, 33, 2076. 22177618 10.1016/j.biomaterials.2011.11.042

[18] J. M. Holzwarth , P. X. Ma , Biomaterials 2011, 32, 9622. 21944829 10.1016/j.biomaterials.2011.09.009 PMC3195926

[19] C. Hu , D. Ashok , D. R. Nisbet , V. Gautam , Biomaterials 2019, 219, 119366. 31374482 10.1016/j.biomaterials.2019.119366

[20] C. G. Knight , L. F. Morton , D. J. Onley , A. R. Peachey , A. J. Messent , P. A. Smethurst , D. S. Tuckwell , R. W. Farndale , M. J. Barnes , J. Biol. Chem. 1998, 273, 33287. 9837901 10.1074/jbc.273.50.33287

[21] C. G. Knight , L. F. Morton , A. R. Peachey , D. S. Tuckwell , R. W. Farndale , M. J. Barnes , J. Biol. Chem. 2000, 275, 35. 10617582 10.1074/jbc.275.1.35

[22] X. Guo , J. Bai , G. Ge , Z. Wang , Q. Wang , K. Zheng , H. Tao , L. Zhang , H. Zhang , D. Wang , X. Zhang , H. Li , G. Pan , D. Geng , J. Colloid Interface Sci. 2022, 605, 410. 34332414 10.1016/j.jcis.2021.07.079

[23] S. Cychy , D. Hiltrop , C. Andronescu , M. Muhler , W. Schuhmann , Anal. Chem. 2019, 91, 14323. 31609106 10.1021/acs.analchem.9b02734

[24] W. Qin , Y. Li , J. Ma , Q. Liang , X. Cui , H. Jia , B. Tang , Dent. Mater. 2020, 36, 1289. 32651018 10.1016/j.dental.2020.06.004

[25] S. Guo , D. Yu , X. Xiao , W. Liu , Z. Wu , L. Shi , Q. Zhao , D. Yang , Y. Lu , X. Wei , Z. Tang , N. Wang , X. Li , Y. Han , Z. Guo , J. Mater. Chem. B 2020, 8, 6048. 32627795 10.1039/d0tb00282h

[26] X. Ren , Y. Feng , J. Guo , H. Wang , Q. Li , J. Yang , X. Hao , J. Lv , N. Ma , W. Li , Chem. Soc. Rev. 2015, 44, 5680. 26023741 10.1039/c4cs00483c

[27] H. Yu , S. Yu , H. Qiu , P. Gao , Y. Chen , X. Zhao , Q. Tu , M. Zhou , L. Cai , N. Huang , K. Xiong , Z. Yang , Bioact. Mater. 2021, 6, 1618. 33294738 10.1016/j.bioactmat.2020.11.011 PMC7695912

[28] G. Pan , S. Sun , W. Zhang , R. Zhao , W. Cui , F. He , L. Huang , S. H. Lee , K. J. Shea , Q. Shi , H. Yang , J. Am. Chem. Soc. 2016, 138, 15078. 27778505 10.1021/jacs.6b09770

[29] H. Lee , S. M. Dellatore , W. M. Miller , P. B. Messersmith , Science 2007, 318, 426. 17947576 10.1126/science.1147241 PMC2601629

[30] Z. Li , Y. Yu , W. Zeng , F. Ding , D. Zhang , W. Cheng , M. Wang , H. Chen , G. Pan , L. Mei , X. Zeng , N. Gao , Small 2022, 18, e2201803. 35616079 10.1002/smll.202201803

[31] X. Zhang , G. Chen , Y. Yu , L. Sun , Y. Zhao , Research 2020, 2020, 3672120. 32490376 10.34133/2020/3672120 PMC7231261

[32] Y. Yang , P. Gao , J. Wang , Q. Tu , L. Bai , K. Xiong , H. Qiu , X. Zhao , M. F. Maitz , H. Wang , X. Li , Q. Zhao , Y. Xiao , N. Huang , Z. Yang , Research 2020, 2020, 9203906. 32405627 10.34133/2020/9203906 PMC7196174

[33] Y. Li , J. Cheng , P. Delparastan , H. Wang , S. J. Sigg , K. G. DeFrates , Y. Cao , P. B. Messersmith , Nat. Commun. 2020, 11, 3895. 32753588 10.1038/s41467-020-17597-4 PMC7403305

[34] M. S. Akram Bhuiyan , J. D. Roland , B. Liu , M. Reaume , Z. Zhang , J. D. Kelley , B. P. Lee , J. Am. Chem. Soc. 2020, 142, 4631. 32046478 10.1021/jacs.9b11266 PMC7068691

[35] J. Bai , H. Wang , H. Chen , G. Ge , M. Wang , A. Gao , L. Tong , Y. Xu , H. Yang , G. Pan , P. K. Chu , D. Geng , Biomaterials 2020, 255, 120197. 32563944 10.1016/j.biomaterials.2020.120197

[36] Y. H. Ding , M. Floren , W. Tan , Biosurf. Biotribol. 2016, 2, 121. 29888337 10.1016/j.bsbt.2016.11.001 PMC5991493

[37] Z. Yang , X. Zhao , R. Hao , Q. Tu , X. Tian , Y. Xiao , K. Xiong , M. Wang , Y. Feng , N. Huang , G. Pan , Proc. Natl. Acad. Sci. USA 2020, 117, 16127. 32601214 10.1073/pnas.2003732117 PMC7368199

[38] J. Kim , C. R. Bertozzi , Angew. Chem. Int. Ed. Engl. 2015, 54, 15777. 26568479 10.1002/anie.201508861 PMC4715665

[39] L. Lin , H. Wang , M. Ni , Y. Rui , T.‐Y. Cheng , C.‐K. Cheng , X. Pan , G. Li , C. Lin , J. Orthop. Transl. 2014, 2, 35.

[40] J. Ma , M. Thompson , N. Zhao , D. Zhu , J. Orthop. Transl. 2014, 2, 118. 10.1016/j.jot.2014.03.004 PMC5044877 27695671

[41] B. Li , P. Thebault , B. Labat , G. Ladam , V. Alt , M. Rupp , C. Brochausen , J. Jantsch , M. Ip , N. Zhang , W. H. Cheung , S. Y. S. Leung , R. M. Y. Wong , J. Orthop. Transl. 2024, 45, 24. 10.1016/j.jot.2023.12.006 PMC10943307 38495742

[42] Z. Huang , Y. Zhang , R. Liu , Y. Li , M. Rafique , A. C. Midgley , Y. Wan , H. Yan , J. Si , T. Wang , C. Chen , P. Wang , M. Shafiq , J. Li , L. Zhao , D. Kong , K. Wang , Biomaterials 2022, 291, 121901. 36356473 10.1016/j.biomaterials.2022.121901

[43] Y. Sun , X. Liu , Y. Zhu , Y. Han , J. Shen , B. Bao , T. Gao , J. Lin , T. Huang , J. Xu , Y. Chai , X. Zheng , ACS Appl. Mater. Interfaces 2021, 13, 59051. 34846853 10.1021/acsami.1c16300

[44] G. Liu , X. Wang , X. Zhou , L. Zhang , J. Mi , Z. Shan , B. Huang , Z. Chen , Z. Chen , Theranostics 2020, 10, 1074. 31938052 10.7150/thno.37931 PMC6956813

[45] A. Donneys , Q. Yang , M. L. Forrest , N. S. Nelson , T. Zhang , R. Ettinger , K. Ranganathan , A. Snider , S. S. Deshpande , M. S. Cohen , S. R. Buchman , npj Regener. Med. 2019, 4, 11. 10.1038/s41536-019-0072-9 PMC6529413 31123600

[46] M. N. Helmus , D. F. Gibbons , D. Cebon , Toxicol. Pathol. 2008, 36, 70. 18337223 10.1177/0192623307310949

[47] A. J. Rao , E. Gibon , T. Ma , Z. Yao , R. L. Smith , S. B. Goodman , Acta Biomater. 2012, 8, 2815. 22484696 10.1016/j.actbio.2012.03.042 PMC3730834

[48] T. de Jong , A. D. Bakker , V. Everts , T. H. Smit , J. Periodontal Res. 2017, 52, 965. 28635007 10.1111/jre.12477

[49] Z. Chen , A. Bozec , A. Ramming , G. Schett , Nat. Rev. Rheumatol. 2019, 15, 9. 30341437 10.1038/s41584-018-0109-2

[50] T. Wang , J. Bai , M. Lu , C. Huang , D. Geng , G. Chen , L. Wang , J. Qi , W. Cui , L. Deng , Nat. Commun. 2022, 13, 160. 35013289 10.1038/s41467-021-27816-1 PMC8748715

[51] W. Liu , J. Li , M. Cheng , Q. Wang , K. W. K. Yeung , P. K. Chu , X. Zhang , Adv. Sci. 2018, 5, 1800749. 10.1002/advs.201800749 PMC6193167 30356934

[52] L. Bai , P. Chen , Y. Zhao , R. Hang , X. Yao , B. Tang , C. Liu , Y. Xiao , R. Hang , Biomaterials 2021, 278, 121162. 34628191 10.1016/j.biomaterials.2021.121162

[53] U. H. Langen , M. E. Pitulescu , J. M. Kim , R. Enriquez‐Gasca , K. K. Sivaraj , A. P. Kusumbe , A. Singh , J. Di Russo , M. G. Bixel , B. Zhou , L. Sorokin , J. M. Vaquerizas , R. H. Adams , Nat. Cell Biol. 2017, 19, 189. 28218908 10.1038/ncb3476 PMC5580829

[54] T. Yuan , M. Tan , Y. Xu , Q. Xiao , H. Wang , C. Wu , F. Li , L. Peng , J. Nanobiotechnol. 2023, 21, 38. 10.1186/s12951-023-01787-5 PMC9896818 36737778

[55] H. Zhu , Z. K. Guo , X. X. Jiang , H. Li , X. Y. Wang , H. Y. Yao , Y. Zhang , N. Mao , Nat. Protoc. 2010, 5, 550. 20203670 10.1038/nprot.2009.238

[56] L. Jiang , W. Zhang , L. Wei , Q. Zhou , G. Yang , N. Qian , Y. Tang , Y. Gao , X. Jiang , Biomaterials 2018, 179, 15. 29960821 10.1016/j.biomaterials.2018.06.035

