# Supplementary material for: Mussel‐Derived and Bioclickable Peptide Mimic for Enhanced Interfacial Osseointegration via Synergistic Immunomodulation and Vascularized Bone Regeneration
Source: Adv Sci (Weinh). 2024 Jun 23;11(32):2401833. doi: 10.1002/advs.202401833 (PMC11348244; doi:10.1002/advs.202401833)
Supplement: Supplementary file 1 — Supporting Information [file ADVS-11-2401833-s001.docx]

**Supporting Information For**

**Mussel-Derived and Bioclickable Peptide Mimic for Enhanced Interfacial Osseointegration via Synergistic Immunomodulation and Vascularized Bone Regeneration**

Wei Zhou ^1,2^, Yang Liu ^2^, Jiale Dong ^2^, Xianli Hu ^2^, Zheng Su ^2^, Xianzuo Zhang ^2^, Chen Zhu ^2,^*, Liming Xiong ^1,^*, Wei Huang ^2,^* , Jiaxiang Bai ^2^*

^1^ Department of Orthopaedics, Union Hospital, Tongji Medical College, Huazhong University of Science and Technology, Wuhan 430022, China. **E-mail:** [xiongliming@hust.edu.cn](mailto:xiongliming@hust.edu.cn) (L. Xiong)

^2^ Department of Orthopaedics, The First Affiliated Hospital of USTC, Division of Life Sciences and Medicine, University of Science and Technology of China, Hefei, 230022, China. **E-mail:** [jxbai1995@ustc.edu.cn](mailto:jxbai1995@ustc.edu.cn) (J. Bai); [zgkdhwei@ustc.edu.cn](mailto:zgkdhwei@ustc.edu.cn) (W. Huang); [zhuchena@ustc.edu.cn](mailto:zhuchena@ustc.edu.cn) (C. Zhu)

**Keywords**: mussel adhesion, tissue adaptation, immunomodulatory, orthopedic implant, bone regeneration


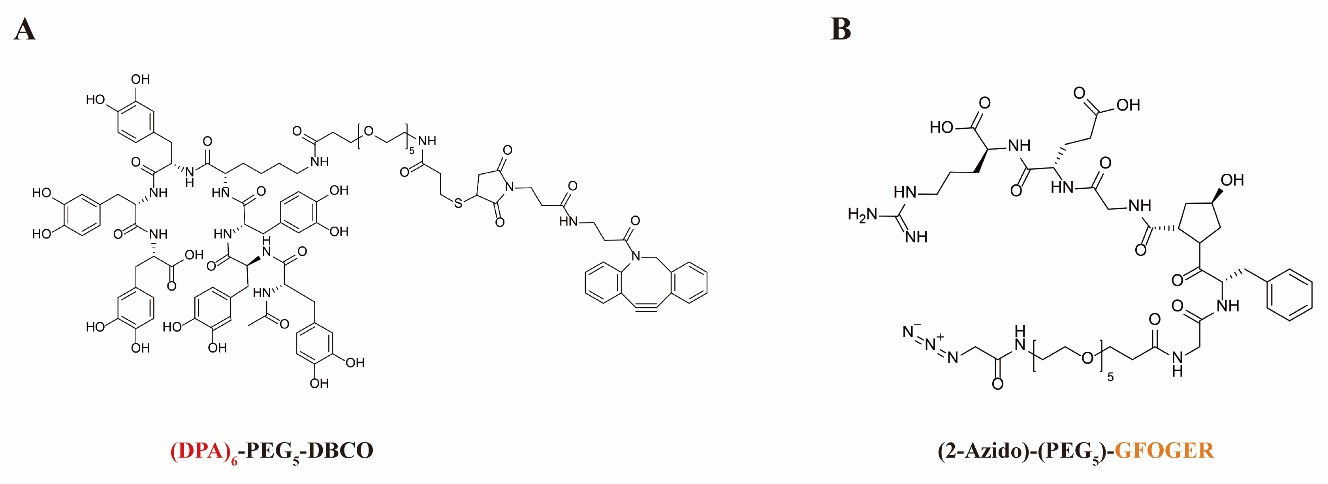
 **Figure S1.** Molecular structures of **(A)** (DOPA)_6_-PEG_5_-DBCO and **(B)** (2-Azido)-PEG_5_-GFOGER.


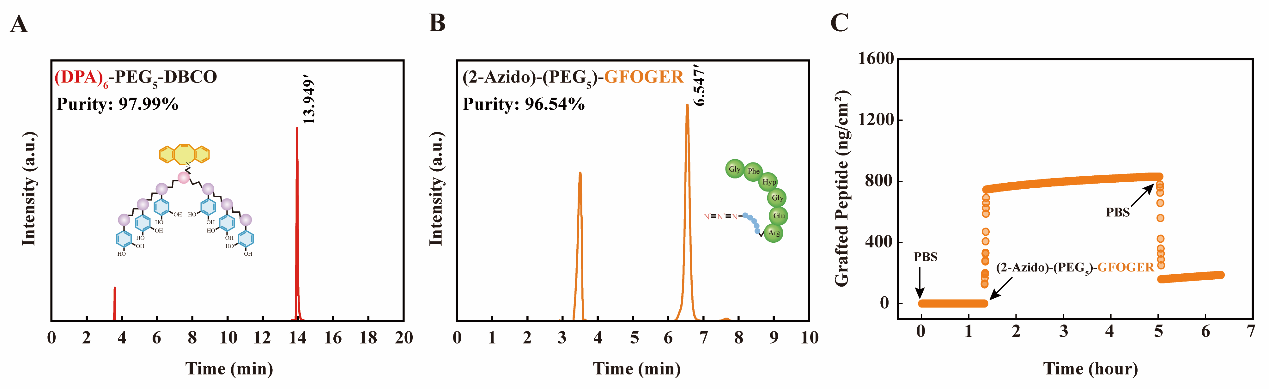


**Figure S2.** HPLC spectra of **(A)** (DPA)_6_-PEG_5_-DBCO and **(B)** (2-Azido)-PEG_5_-GFOGER with purity >96%. **(C)** Real-time monitoring of the binding of the (2-Azido)-PEG_5_-GFOGER cografting process on the (DPA)_6_-PEG_5_-DBCO-bound chips.


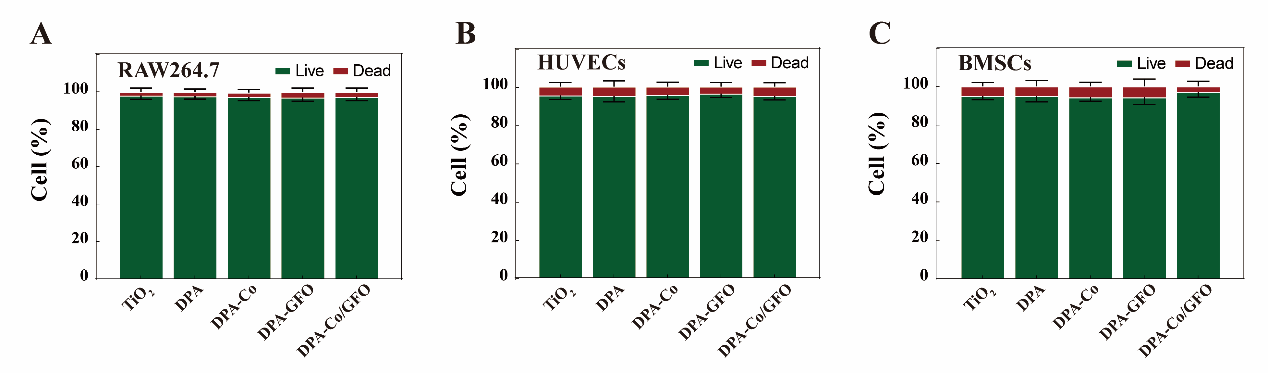


**Figure S3.** Quantitative data from live/dead cell staining of RAW264.7 cells, HUVECs, and BMSCs.


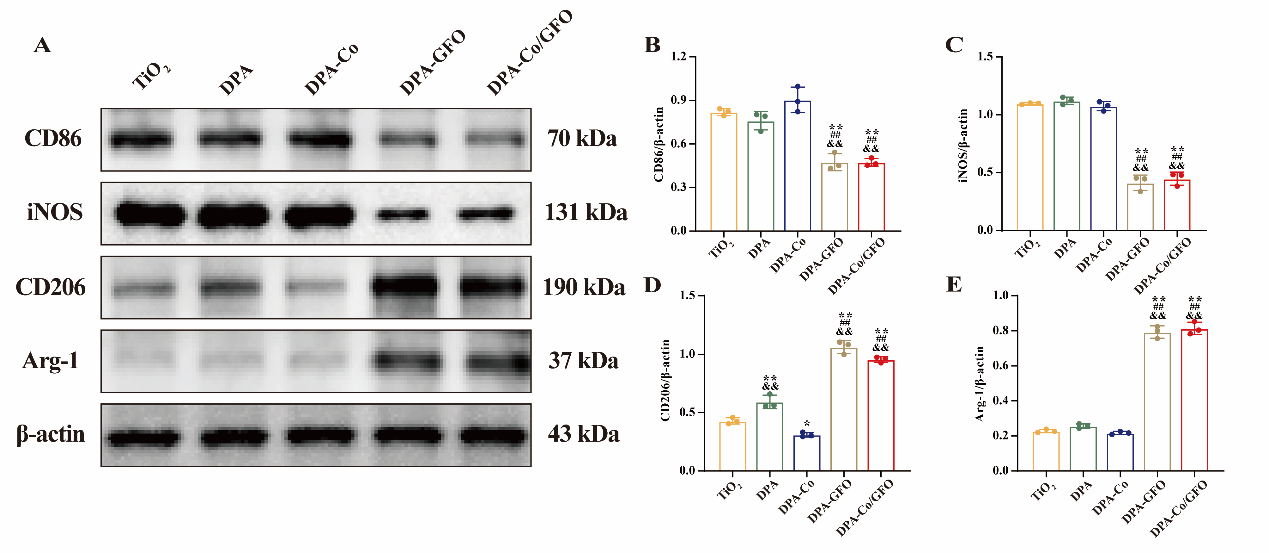


**Figure S4. (A)** Western blot analysis of macrophage polarization-related proteins (M1: CD86 and Inos; M2: CD206 and Arg-1) and **(B-E)** the quantitative results. The data are presented as the mean ± standard deviation (SD); n = 3 per group. ​Statistical analysis was performed by one-way ANOVA; ^∗^P < 0.05 and ^∗∗^P < 0.01 vs. the TiO_2_ group; ^#^P < 0.05 and ^##^P < 0.01 vs. the DPA group; ^&^P < 0.05 and ^&&^P < 0.01 vs. the DPA-Co group.


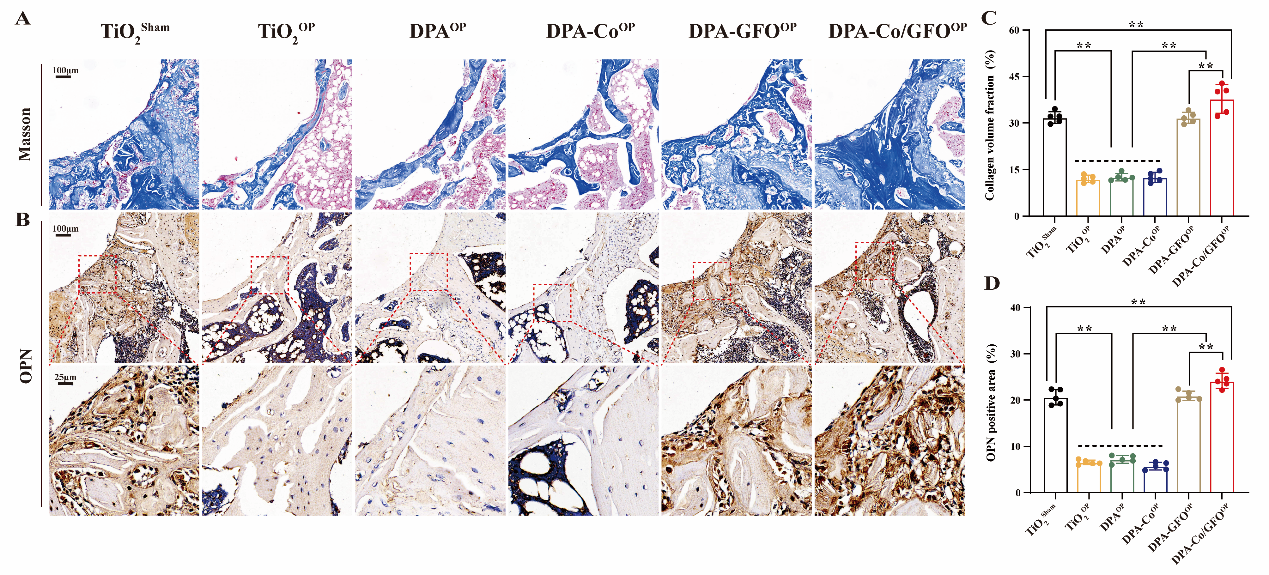


**Figure S5. (A)** Masson staining of the peri-implant tissue in the femur 2 months after implantation and **(C)** the quantitative results. **(B)** Immunohistochemical staining of peri-implant tissue to examine osteogenic-related protein (OPN) expression and **(D)** the quantitative results. The data are presented as the mean ± standard deviation (SD) (n=5 per group). ​Statistical analysis was performed by one-way ANOVA, and *P < 0.05 and **P < 0.01 indicate statistical significance.


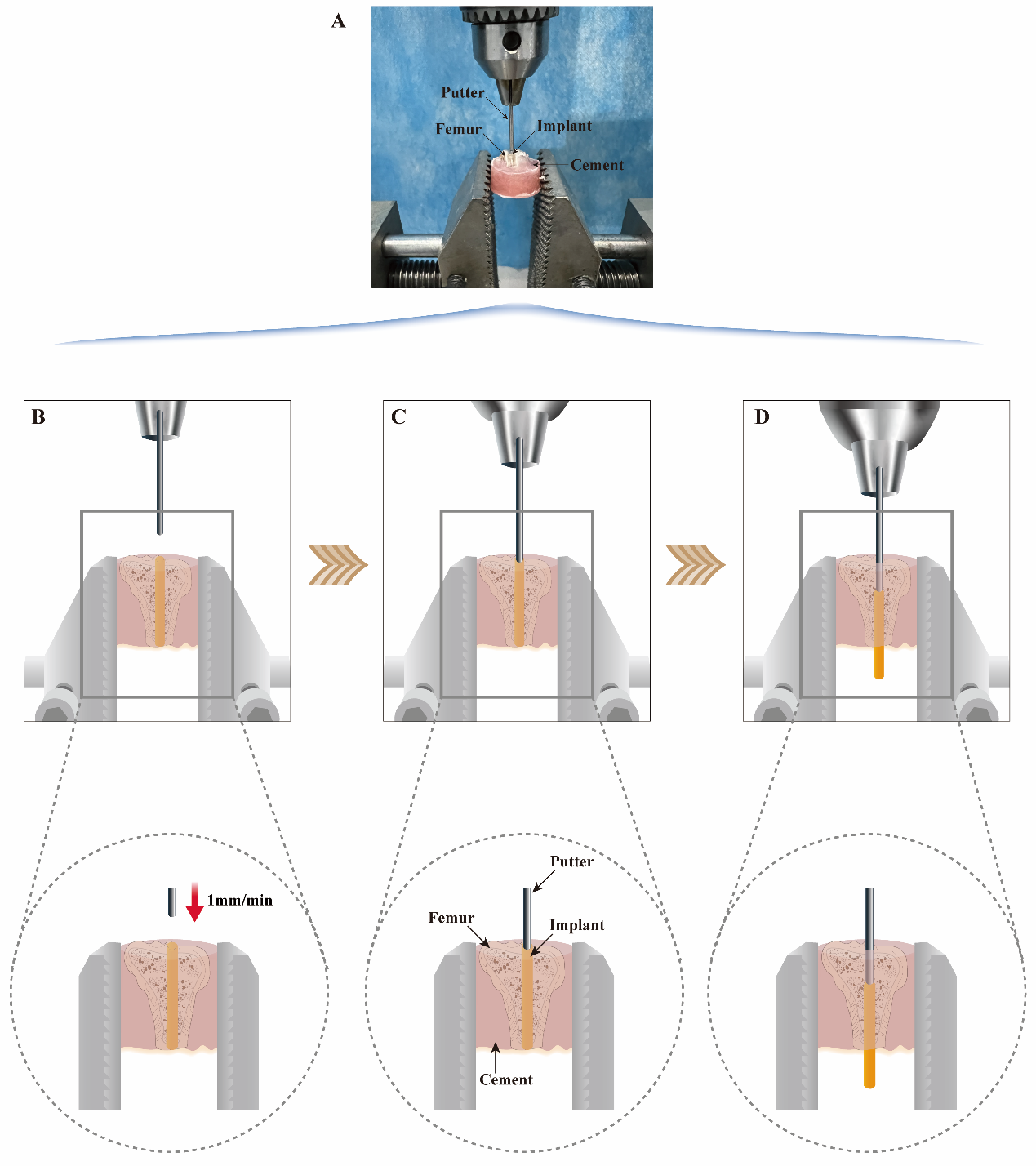


**Figure S6. (A)** Real picture of the implant push-out test. **(B-D)** Flow chart of the implant push-out test.


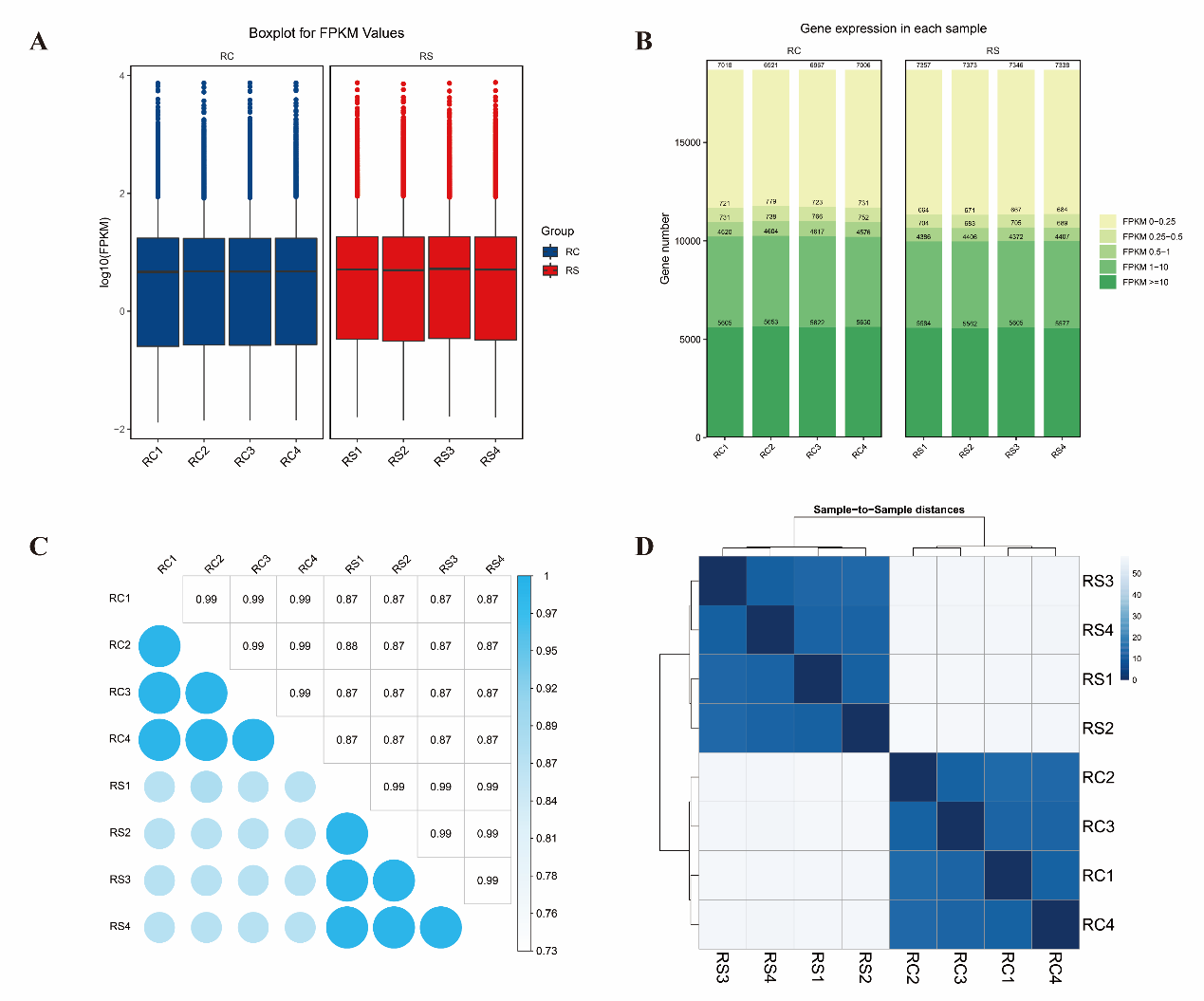


**Figure S7. (A)** Boxplot of gene expression levels and **(B)** the regional distribution chart. **(C)** Heatmap of intersample correlation coefficients. **(D)** Results of sample-to-sample cluster analysis.


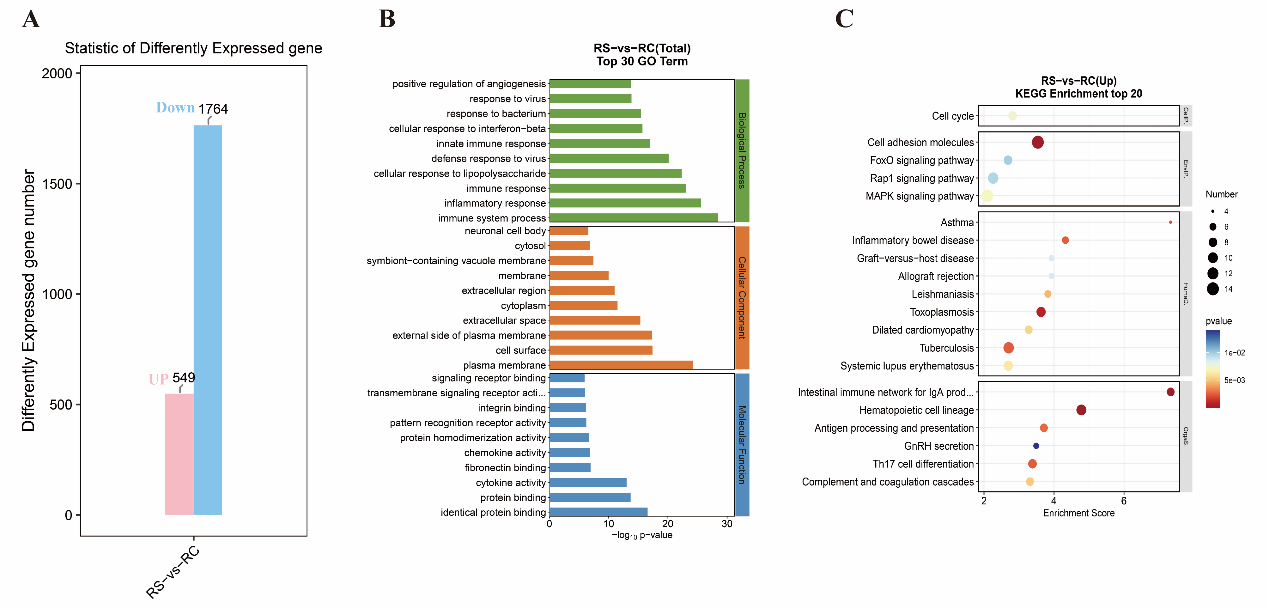


**Figure S8. (A)** Differential gene expression statistical bar chart. **(B)** Results of the GO enrichment analysis. **(C)** KEGG enrichment in the top 20 pathways (top).

**
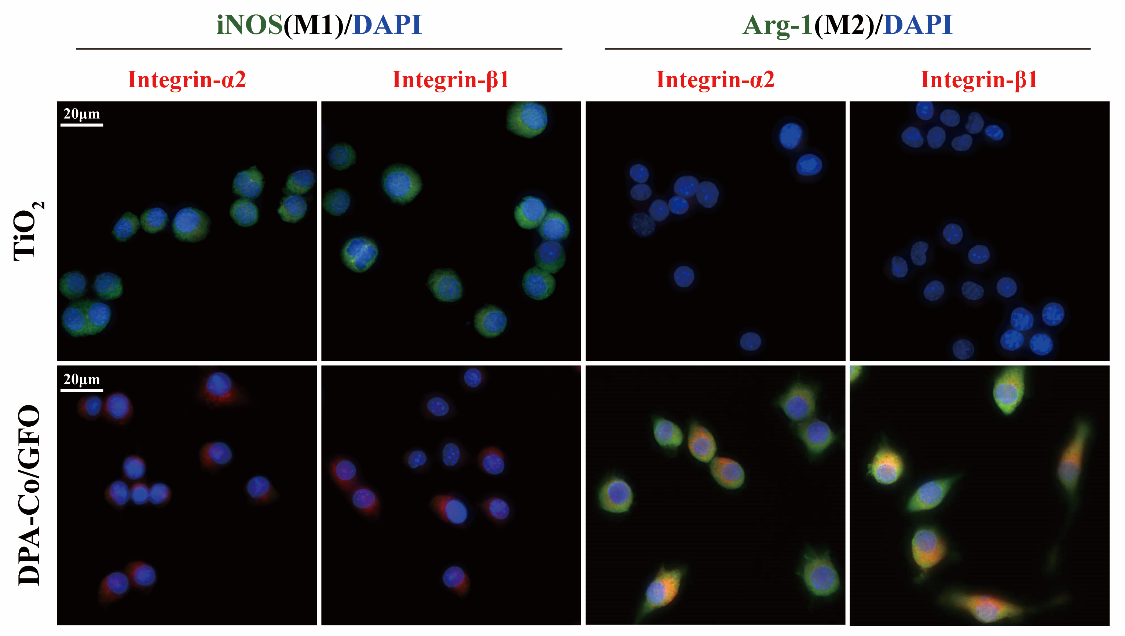
**

**Figure S9.** Fluorescence double-staining images show integrin receptor α2β1 (red) and macrophage polarization markers (green, M1: iNOS, M2: Arg-1).


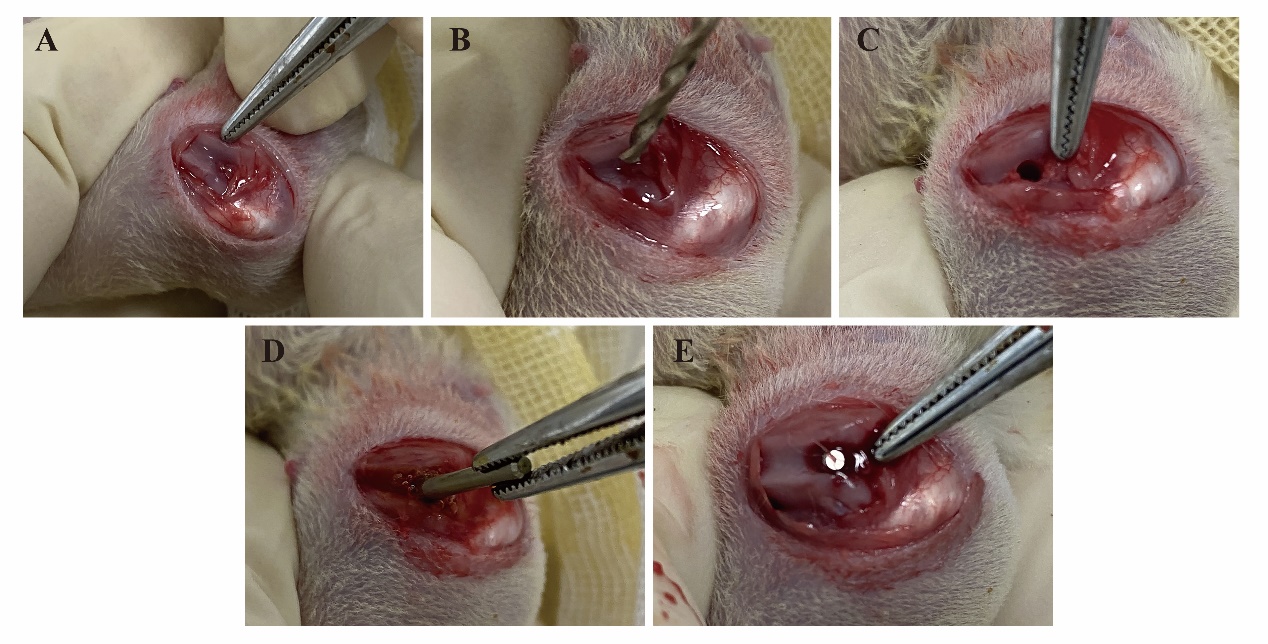


**Figure S10. (A)** Exposure of the femur condyles. **(B)** Drilling of the femur condyles. **(C)** Completion of the drilling process. **(D)** Implantation of the titanium rod. **(E)** Completion of the implantation process.
